# Biological, psychological and social processes that explain celebrities’ influence on patients’ health-related behaviors

**DOI:** 10.1186/2049-3258-73-3

**Published:** 2015-01-19

**Authors:** Steven J Hoffman, Charlie Tan

**Affiliations:** Faculty of Law, University of Ottawa, Ottawa, Canada; Department of Clinical Epidemiology & Biostatistics and McMaster Health Forum, McMaster University, Hamilton, Canada; Department of Global Health & Population, Harvard School of Public Health, Boston, MA USA; Michael G. DeGroote School of Medicine, McMaster University, Hamilton, Canada

**Keywords:** Medical advice, Celebrities, Health literacy, Evidence-based medicine

## Abstract

**Background:**

Celebrities can have substantial influence as medical advisors. However, their impact on public health is equivocal: depending on the advice’s validity and applicability, celebrity engagements can benefit or hinder efforts to educate patients on evidence-based practices and improve their health literacy. This meta-narrative analysis synthesizes multiple disciplinary insights explaining the influence celebrities have on people’s health-related behaviors.

**Methods:**

Systematic searches of electronic databases BusinessSource Complete, Communication & Mass Media Complete, Humanities Abstracts, ProQuest Political Science, PsycINFO, PubMed, and Sociology Abstracts were conducted. Retrieved articles were used to inform a conceptual analysis of the possible processes accounting for the substantial influence celebrities may have as medical advisors.

**Results:**

Fourteen mechanisms of celebrity influence were identified. According to the economics literature, celebrities distinguish endorsed items from competitors and can catalyze herd behavior. Marketing studies tell us that celebrities’ characteristics are transferred to endorsed products, and that the most successful celebrity advisors are those viewed as credible, a perception they can create with their success. Neuroscience research supports these explanations, finding that celebrity endorsements activate brain regions involved in making positive associations, building trust and encoding memories. The psychology literature tells us that celebrity advice conditions people to react positively toward it. People are also inclined to follow celebrities if the advice matches their self-conceptions or if not following it would generate cognitive dissonance. Sociology explains how celebrities’ advice spreads through social networks, how their influence is a manifestation of people’s desire to acquire celebrities’ social capital, and how they affect the ways people acquire and interpret health information.

**Conclusion:**

There are clear and deeply rooted biological, psychological and social processes that explain how celebrities influence people’s health behaviors. With a better understanding of this phenomenon, medical professionals can work to ensure that it is harnessed for good rather than abused for harm. Physicians can discuss with their patients the validity of celebrity advice and share more credible sources of health information. Public health practitioners can debunk celebrities offering unsubstantiated advice or receiving inappropriate financial compensation, and should collaborate with well-meaning celebrities, leveraging their influence to disseminate medical practices of demonstrated benefit.

**Electronic supplementary material:**

The online version of this article (doi:10.1186/2049-3258-73-3) contains supplementary material, which is available to authorized users.

## Background

Celebrities frequently give medical advice and people often follow it. Whether motivated by good intentions or financial compensation, celebrity endorsements can generate large publicity for health campaigns by virtue of the spokespersons’ visibility, public interest and perceived newsworthiness. When journalist Katie Couric televised her colonoscopy on NBC’s Today Show in 2000, colorectal cancer screenings by 400 American endoscopists increased by 21% the next month [1]. Following actor-singer Kylie Minogue’s diagnosis with breast cancer, mammography bookings rose 40% in four Australian states [2]. Twice as many cervical cancer screenings were conducted in England during March 2009 as compared to the same month one year earlier, corresponding to reality TV’s Jade Goody passing from the disease [3].

Although few in number, empirical studies have also shown the considerable influence celebrities can have. A 2009 survey of 1,552 Americans found that 24% of parents place “some trust” in vaccine safety information given by celebrities [4]. Both parents and children are more likely to choose food products endorsed by celebrities, with one study finding that children who viewed a celebrity endorsement or even footage of the endorser in a different context subsequently consumed greater quantities of the endorsed item [5–7].

Celebrity health engagements are not new developments or transient fads. Rather, there is a long history of celebrities giving medical advice. In 1999, American politician Bob Dole collaborated with Pfizer to raise awareness about erectile dysfunction, and a year later actor Julie Andrews starred in a television advertisement for the osteoporosis drug Evista. More recently, singer Adam Levine worked with Shire to raise awareness for ADHD, and actor Sally Fields starred in advertisements for Boniva, an osteoporosis medication for postmenopausal women. The ubiquitous nature of modern media and the advent of new communication technologies mean that celebrity advice can spread far wider and more rapidly than ever before, making its influence increasingly pervasive and powerful.

Among today’s celebrity medical advisors, many have mobilized their influence for good. The Michael J. Fox Foundation for Parkinson’s Research has raised over $350 million USD for research seeking a cure for Parkinson’s disease [8]. Singer Sir Elton John is a highly dedicated AIDS advocate; his foundation has raised more than $300 million USD to fight HIV/AIDS [9]. But the messages espoused by celebrities can also be at odds with those from health professionals, public health authorities and the best available research evidence. British television presenter Sir Michael Parkinson promoted an unsupported (and potentially harmful) self-diagnosis technique for prostate cancer: “The test is if you can pee against a wall from two feet, you haven't got it” [10]. Having breast cancer at age 36, actor Christina Applegate supported MRI screening for early detection; yet advisory groups do not endorse MRIs for individuals at average breast cancer risk [11]. In May 2013, singer Katy Perry tweeted a photo of herself with three large bags of pills, one for each daily meal, with the caption, “I’m all about that supplement and vitamin LYFE!” What Perry withheld from her 63 million Twitter followers was that, based on numerous systematic reviews [12–16], a 2013 editorial in the *Annals of Internal Medicine* definitively recommended against non-prescribed vitamin and mineral supplements for chronic disease prevention [17]. Actor Suzanne Somers advocates her own brand of medicine, including bioidentical hormones to reverse aging and proteolytic enzyme therapy for pancreatic cancer, despite a lack of supporting evidence [18, 19]. Likewise, Jenny McCarthy warns about a link between vaccinations and autism, a wholly discredited claim that is thought to be partially responsible for recent outbreaks of vaccine-preventable diseases in North America and the United Kingdom [20–23].

Celebrities can thus act as powerful public health tools, agents who disseminate and encourage health behaviors of proven benefit. However, their influence becomes deeply troubling when their medical advice is uninformed and possibly dangerous. For example, following Parkinson’s prostate cancer test could discourage men from seeking proper medical diagnosis. Applegate’s favored breast MRIs cost over $1,000 USD, approximately ten times more than a mammography [11]. A randomized controlled trial comparing chemotherapy and Somers-endorsed proteolytic enzyme therapy for pancreatic cancer found the former offered significantly longer survival times and higher life quality [19].

This meta-narrative analysis takes an interdisciplinary approach to examine how celebrities become trusted medical advisors and why the public often follows their advice when making health decisions. We have updated and expanded upon previous systematic searches of the economics, marketing, psychology, and sociology literatures [24], added new insights from a systematic search of the neuroscience literature, and integrated insights from across the five disciplines with additional targeted searches to explore why celebrities have influence on people’s health-related behaviors.

## Methods

Electronic databases of research literature for each discipline were identified and selected in consultation with a specialized social science librarian. The generic search phrase used for each database was: (celebrity OR “famous people” OR “famous person*” OR “star”) near/5 (advert* OR advocat* OR campaign OR diet* OR endors* OR market* OR produc* OR promot*). Minor adjustments were made to this phrase to account for syntax differences among databases. Additional limitations were used for some databases to optimize the search for relevant results (see Table 1). Systematic searches were performed between January 11–21, 2013, except the search for sociology literature, which was conducted separately on June 13, 2013. Additional targeted searches were conducted until March 2014.

**Table 1:** **Detailed search protocol**

Field	Database and justification	Search phrase	Number yielded	Number included ^a^
**Medical**	PubMed, 1966-2013	(celebrity OR “famous people*” OR “famous person*”) AND (endors* OR campaign* OR advert* OR advocat* OR diet* OR promot* OR market* OR produc*)^b^	447	14
	*Most comprehensive database of the medical literature.*			
**Marketing/Business**	Business Source Complete, 1886-2013	(celebrity OR “famous people” OR “famous person*” OR star) N5 (advert* OR advocat* OR campaign OR diet* OR endors* OR market* OR produc* OR promot*)^c^	461	14
	*Emphasizes scholarly journals over trade publications; contains more scholarly journals than ABI/INFORM Complete.*			
**Communications**	Communication and Mass Media Complete, 1915-2013	(celebrity OR “famous people” OR “famous person*” OR star) N5 (advert* OR advocat* OR campaign OR diet* OR endors* OR market* OR produc* OR promot*)^d^	213	14
	*Contains more scholarly journals than main competitor, Communication Abstracts.*			
**Psychology**	PsycINFO, 1806-2013	ALL((celebrity OR “famous people” OR “famous person*” OR star) n/5 (advert* OR advocat* OR campaign OR diet* OR endors* OR market* OR produc* OR promot*))	333	19
	*Primary database for literature in psychology, with over 1800 scholarly journals.*			
**Culture**	Humanities Abstracts, 1984-2013	(celebrity OR “famous people” OR “famous person*” OR star) N5 (advert* OR advocat* OR campaign OR diet* OR endors* OR market* OR produc* OR promot*)	166	2
	*No dedicated cultural studies databases exist; therefore the most comprehensive humanities database was selected.*			
**Political Science**	ProQuest Political Science, 1985-2013	ALL((celebrity OR “famous people” OR “famous person*” OR star) n/5 (advert* OR advocat* OR campaign OR diet* OR endors* OR market* OR produc* OR promot*))	327	0
	*Combined searching of PAIS International and Worldwide Political Science Abstracts, the two largest databases in this field.*			
**Neuroscience**	PubMed, 1966-2013	(celebrity OR “famous people*” OR “famous person*”) AND (brain OR fMRI OR neuro* OR neural)	419	9
	*Most comprehensive database of the medical literature*.			
**Sociology**	Sociology Abstracts, 1952-2013	ALL((celebrity OR “famous people” OR “famous person*” OR star) n/5 (advert* OR advocat* OR campaign OR diet* OR endors* OR market* OR produc* OR promot*))	194	32
	*Primary database for literature in sociology, with over 1800 periodicals.*			

^a^Documents were included if they were both relevant to the study and unique from those found in earlier searches of different databases. This table presents the databases in the order they were searched. ^b^The word “star” was not included in the search strategy for PubMed due to prevalence of the word in medical research (e.g., “StAR” is an acronym for steroidogenic acute regulatory protein). ^c^The source type was limited to academic journals to filter out trade publications. ^d^The source type was limited to academic journals to filter out trade publications. The asterisk sign was used in the search phrases to search for word variations (e.g., searching person* returns person and personality).

Titles and abstracts were assessed to identify studies for inclusion. To be included in the review, retrieved documents had to discuss celebrity influence, either within or outside of an endorsement context. The full texts were then read to identify relevant mechanisms accounting for the substantial influence celebrities may have as medical advisors. Mechanisms were selected based on the number of supporting studies as well as the quality of the studies, with articles that reported experimental evidence, empirical findings or well-established theories prioritized. Pertinent information from these studies, including authors, year of publication, methodology and key findings, was extracted to inform a conceptual analysis of the possible mechanisms. One author (CT) conducted the literature searches, and both authors reviewed summaries of results to identify the mechanisms that were most helpful in explaining celebrities’ influence. Additional targeted searches were conducted to supplement the analyses and allow for a more comprehensive overview of the identified insights. Additional file 1 presents the detailed search results by discipline.

## Results

Our systematic searches of the economics, marketing, neuroscience, psychology, and sociology literatures yielded 2560 publications, resulting in 104 separate studies on celebrity influence. From these studies as well as studies retrieved from additional targeted searches, we identified 14 potential mechanisms through which celebrities may influence people’s health decisions (see Table 2).

**Table 2:** **Mechanisms explaining celebrity influence**

Discipline	Mechanism	Description
Economics	1) Signals	Celebrity endorsements act as markers that differentiate endorsed items from competitors.
	2) Herd behavior	Celebrities activate people’s natural tendency to make decisions based on how others have acted in similar situations.
Marketing	3) Meaning transfer	People consume items to acquire the endorsing celebrities’ traits, which have become associated with the product.
	4) Source credibility	Celebrities share personal experiences and success stories associated with the endorsed item to be perceived as credible sources of health information.
	5) Halo effect	The specific success of celebrities is generalized to all their traits, biasing people to view them as credible medical advisors.
Neuroscience	6) Neural mechanisms of meaning transfer	Celebrity advertisements activate a brain region involved in forming positive associations, indicating the transfer of positive memories associated with the celebrity to the endorsed item.
	7) Neuropsychology of credibility	Endorsements from celebrities activate brain regions associated with trustful behavior and memory formation, thereby improving attitudes toward and recognition of the endorsed item.
Psychology	8) Classical conditioning	The positive responses people have toward celebrities come to be independently generated by endorsed items.
	9) Self-conception	People follow advice from celebrities who match how they perceive (or want to perceive) themselves.
	10) Cognitive dissonance	People unconsciously rationalize following celebrity medical advice to reduce the psychological discomfort that may otherwise result from holding incompatible views.
	11) Attachment	People, especially those with low self-esteem, form attachments to celebrities who make them feel independent in their actions, supported by others, and competent in their activities.
Sociology	12) Social networks	Celebrity advice reaches large masses by spreading through systems of people linked through personal connections.
	13) Commodification and social capital	People follow celebrity medical advice to gain social status and shape their social identities.
	14) Social constructivism	Celebrity medical advice may alter how people perceive health information and how it is produced in the first place.

### Signals (economics)

When celebrities endorse a product or idea, they differentiate it from others. According to *signaling theory*, “signals” are tools that convey key information about an object or individual, and are interpreted by the receiver to aid in making a decision [25]. Consumers of health information are often overwhelmed by contradicting advice from health professionals, friends, family and online resources. Given the array of recommended health products and behaviors, choosing among these options and making informed health decisions is difficult. To help in this task, people naturally look for “signals” that indicate one option as being more credible and effective than others [25]. Due to the vaulted status of celebrities in society, their endorsements act as signals of superiority that distinguish the endorsed item from competitors, nudging people to change their health behaviors accordingly.

### Herd behavior (economics)

The influence of celebrities’ medical advice is strengthened by people’s natural tendency to make decisions based on what others have done in similar situations [26]. There are various reasons for this inclination to imitate, including the safety of numbers, comfort in adopting accepted opinions, and desire to acquire what others have [27]. Viewed as trendsetters, celebrities are often early leaders of *herd behaviors*, whether involving new diets, exercise routines or medical procedures [28]. Wanting to follow in their favorite celebrities’ footsteps, many will ignore their personal information and imitate the celebrity health choices they observe [26]. This behavior initiates an informational cascade: the celebrity’s decisions are passed to others who make the same choices [28]. As the number of followers increases, the herding effect lengthens and strengthens, spreading from person-to-person and changing health behaviors along the way [28]. For instance, actor Angelina Jolie’s announcement that she had undergone a preventive double mastectomy after testing positive for the BRCA1 gene mutation led to explosive interest in genetic testing [29]. However, due to the low prevalence of BRCA mutations, a recent systematic review only recommends testing in women who have family members with BRCA-associated cancers and who have had appropriate genetic screening and counselling [30]. Even though routine testing is not recommended for women without a family history of BRCA mutations, Jolie’s announcement may have catalyzed a herd of women seeking the test, including many for whom it is neither appropriate nor cost-effective.

### Meaning transfer (marketing)

Celebrities may be successful medical advisors because consumers see in them attributes they respect and want to emulate. This desire stems from a process marketing researchers call *meaning transfer*. For many people, celebrities represent important social or cultural meanings that become associated with ideas or products they endorse [31]. People will in turn consume these items in hopes of acquiring the celebrities’ traits [32]. The tobacco industry has a history of using this process to sell their products. Through fostering close relationships with movie studios and prominently featuring stars in advertisements [33, 34], companies try to transfer the attractive and sophisticated image of celebrities to their cigarettes. The strategy works: smoking in movies has been found to alter perceptions of and susceptibilities toward smoking among adolescents [35]. In turn, such youths are more likely to initiate cigarette use and continue smoking behavior into adulthood [36–39]. Similarly, food and beverage companies have recruited sports celebrities to endorse their often unhealthy products, with one marketing analysis finding that such items were the second most frequently endorsed by athletes [40]. Of these food products, 79% were classified as energy-dense and nutrient poor, while 93% of endorsed beverages derived 100% of their calories from additional sugars [40]. The healthful impression people have toward professional athletes become transferred to the endorsed products, with one experimental study finding that parents perceive foods endorsed by athletes to be healthier even if they are not [7].

The same process occurs when celebrities attribute their good health, beauty and vitality to particular practices. Following a period of fatigue, anemia and vitamin D deficiency, actor Gwyneth Paltrow credited a three-week elimination diet for her recovery. By abstaining from various foods to which she was sensitive, Paltrow “cleansed” her body and came to lose weight, look better and feel more energetic. This diet, promoted in her latest cookbook [41], is not supported by research evidence; indeed, elimination diets are primarily studied to treat severe allergies and food intolerances [42, 43], and testing for food sensitivity is unfounded and expensive [44, 45]. Nevertheless, Paltrow’s slender physique, youthful appearance and healthful being, meanings transferred to her elimination diet, may convince people to adopt the diet without considering the potential risks or consulting their physician.

### Source credibility (marketing)

The most successful celebrity medical advisors are those perceived to have high credibility. According to the *source credibility model*, credibility depends on trustworthiness and expertise [46]. Trustworthiness refers to how honest and believable the endorser is in giving an opinion on the product [47], while expertise is the extent to which an endorser is thought to be a valid source of information [48]. Credibility also creates congruence between celebrities and the items they promote, with studies finding that advertisements are more successful when the endorser’s image matches the pertinent attributes of the product [49, 50]. In acting as medical advisors, celebrities have, or portray themselves to have, an authentic connection [51]. Many promote health behaviors for conditions they have personally suffered, such as former basketball player Magic Johnson’s endorsement of the home HIV test OraQuick [52] and actor Brooke Shields’ promotion of Paxil to treat postpartum depression [53]. Others endorse products they credit for achieving their admired or respected traits. Numerous celebrities, including actor-singers Katy Perry, Jessica Simpson and Justin Bieber, have shared their troubles with acne and credited Proactiv Solution for their now clear complexions [54]. After singer Carnie Wilson live-broadcast her gastric bypass surgery, and later made the media rounds to tout the procedure for her new frame, the number of individuals undergoing the surgery in the US increased from 19,000 to 100,000 per year [55]. By sharing their past experiences and the sources of their health achievements, these celebrities are perceived as credible, enticing people to follow their advice.

### Halo effect (marketing)

However, even celebrities without a genuine connection have been perceived as credible health advisors. This false medical credibility may stem from difficulty in separating it from a more generalized impression of the celebrity. In the *halo effect*, the predominant trait of an individual biases how all his or her other traits are judged [56]. People exceptional in one way are assumed to similarly excel in other areas [56]. The public often associates the perceived success of celebrities with generalized trustworthiness and wide-reaching expertise that extends beyond the celebrities’ industry and skill set. Celebrities are in turn perceived to have greater credibility than their non-celebrity counterparts, like physicians, despite having less medical knowledge and experience. Actor Lauren Bacall’s endorsement of Visudyne was founded on an unnamed friend’s experience with macular degeneration [57]. Food personality Paula Deen sponsored the diabetes medication Victoza despite how her reputation as the “Butter Queen” would put her trustworthiness into question [58]. Even though celebrities are often compensated for their endorsements, their riches and achievements pull consumers to view them as credible and follow their health recommendations.

### Neural mechanisms of meaning transfer (neuroscience)

The influence of celebrity health endorsements may involve distinct cognitive processes. One study involving 26 women provides evidence for a mechanism similar to meaning transfer. When participants were presented with shoes paired with a celebrity’s face – paralleling an advertisement – functional magnetic resonance imaging (fMRI) scans found their *medial orbitofrontal cortex* was activated [59]. The orbitofrontal cortex is involved in connecting neutral and reinforcing stimuli, with the medial portion specifically encoding positive associations [60]. Therefore, activation of this region may indicate transfer of positive attributes from the celebrity (the reinforcing stimulus) to the object (the neutral stimulus) [59]. Brain regions associated with explicit memories were also activated, suggesting that the transferred meanings stem from conscious recollections of past facts and episodes associated with the celebrity, rather than unconscious implicit memories [59]. When people view celebrity health endorsements, they retrieve explicit memories related to the celebrity. If the memories are positive, they are transferred to the product or idea being endorsed and promote its adoption.

### Neuropsychology of credibility (neuroscience)

There may also be a neural basis for credibility. It relates to the effect of celebrities’ perceived expertise – a component of the source credibility model from marketing literature – on the persuasiveness of endorsements. One study found consumers’ purchase intention and product recognition were increased for products accompanied by “expert celebrities” as compared to “non-expert celebrities” [61]. This indicated that celebrity expertise has long-term positive effects on consumers’ attitude toward and ability to recall brands and products. fMRI scans revealed that seeing expert celebrities activated the *caudate nucleus*, a subcortical region involved in promoting trustful behavior and processing risks and rewards. Celebrities with high expertise may thus promote favorable attitudes toward the endorsed item by inducing trust and leading people to reassess the item’s value. In addition, memory formation was shaped at the *medial temporal lobe*, which is involved in memory encoding. Areas associated with understanding concepts and meanings were also activated. This suggests that increased processing of already-learned celebrity and product information causes the medial temporal lobe to create a favorable and lasting memory of the endorsed item, thereby encouraging its consumption [61]. Although activation of brain regions on fMRI is only a surrogate marker of underlying neural mechanisms and the unique functions of individual structures are difficult to separate, these studies indicate that innate biological processes may exist that cultivate people’s trust in celebrities as medical advisors.

### Classical conditioning (psychology)

Human psychology may also explain the substantial influence celebrities have on health decisions. *Classical conditioning* is a process by which people learn to associate two stimuli such that exposure to either achieves similar responses [62]. Celebrities, in this case, are unconditional stimuli that elicit positive unconditional responses. Through repeat pairings over time, the things celebrities endorse come to elicit a positive conditional response given their association with the celebrity. These items become conditional stimuli whereby they elicit the same positive sentiments even without the celebrity. One recent study found that coupling an attractive and trustworthy celebrity with a product as an unconditional-conditional stimulus pairing led to significantly higher product ratings, indicating a positive conditional response [62].

In addition, according to the concept of belongingness in conditioning research, a conditional stimulus that is closely matched with an unconditional stimulus more easily evokes the conditional response [62]. High congruency between celebrities and their medical advice should thus lead to more intense sentiments generated toward the message. In one study, pairing a product with a highly congruent celebrity as compared to a poorly congruent one led to stronger conditioning, in the form of a more positive attitude [62]. Therefore, celebrity medical advice may be conditioned to evoke consumers’ positive perceptions of celebrities, an effect that is strengthened when the advice matches the celebrity’s image.

### Self-conception (psychology)

The psychology literature also suggests that advice from celebrities who match people’s self-conception have greater influence. *Self-conception* includes the thoughts and attitudes people have of their actual self, those they would like for their ideal self, and those they use to present their social self [63]. Just as how products or brands are marketed, celebrities create an image for themselves they hope resonates with their fans [31]. Since people frequently use these images to define their self-conception, congruent advice can be highly effective [64, 65]. For celebrities viewed as inspirational and personally relevant, their advice will be compatible with people’s ideal self such that the *self-esteem motive* – to elevate one’s actual self toward one’s ideal self [64] – pushes people to follow the advice. One study found that compatibility between a celebrity endorser’s image and a person’s ideal self was associated with higher advertisement ratings and greater purchase intention [66]. Conversely, for celebrities who portray themselves as similar to their admirers, their advice will be compatible with people’s actual self such that the *self-consistency motive* – to maintain one’s actual self [64] – is the motivating factor.

### Cognitive dissonance (psychology)

The desire to maintain mental consistency may account for why people follow celebrity medical advice. According to *cognitive dissonance theory* from social psychology, people experience psychological discomfort, or dissonance, when there is conflict between the decisions they make, the behaviors they choose, the information they hear, and/or the beliefs, opinions, values and ideas they hold [67]. This discomfort will naturally motivate people to reduce or avoid dissonance [67]. Cognitive dissonance has been used to explain how people rationalize difficult decisions [68]. For example, when celebrities offer medical advice, ardent fans may experience dissonance if they do not follow it: the action conflicts with their celebrity adoration. However, following the advice can also create discomfort since endorsed behaviors are often difficult, expensive and unconventional. To reduce dissonance, followers unconsciously modify their cognitions, such as believing the celebrity advice is more credible than alternatives [69]. They also adopt new beliefs or commit to actions that diminish inconsistencies, including seeking information supporting the celebrity advice [69]. Lastly, they trivialize dissonant cognitions to make the conflict seem less important, such as minimizing the advice’s costs and harms [69]. In this way, people justify their decisions to follow celebrities’ medical advice while strengthening their celebrity attachments in the process.

### Attachment (psychology)

People are prone to celebrity influence if they have strong feelings of *attachment* toward a celebrity. Previous research has demonstrated that intense attachments can result if celebrities are responsive to people’s needs for autonomy, relatedness and competence. Autonomy is the need to believe that one’s actions are self-determined without constraint or coercion. Relatedness is the need to feel intimate with and cared for by others. Competence is the need to feel effective and capable in one’s activities [70]. Celebrities can provide inputs to fulfill these needs, thereby fostering strong attachments. For example, media queen Oprah Winfrey has built a legion of ardent fans, in part through such inputs. Through her numerous outlets, she urges viewers to take control of their own lives, shares personal details and emotions, and encourages people to feel confident and worthy. Given their strong attachments, many of Oprah’s followers faithfully adopt her sometimes-dubious medical advice. When Oprah promoted the herbal cold remedy Airborne on her television show, sales of the supplement soared despite a lack of supporting research evidence. Airborne’s manufacturers claimed that a double-blind, placebo-controlled study supported the supplement’s effectiveness; however, the company that conducted the study, GNG Pharmaceutical Services, turned out to be a two-man enterprise with no scientists or doctors, and created just to perform the Airborne “study” of questionable validity [71]. The U.S. Federal Trade Commission even charged Airborne’s manufacturers in 2008 for falsifying claims of efficacy, eventually reaching a $30 million USD settlement [72]. However, Oprah’s followers and others continue to buy and use the product [71].

Having a poor sense of self-identity and/or low self-esteem also makes people more susceptible to celebrity attachment [73]. In three studies on parasocial interactions – unidirectional connections fans make with media personalities – people with low self-esteem used celebrity relationships to move closer to their ideal self, a benefit that people with high self-esteem derive from real relationship partners [74]. Two models can explain this tendency. In the *absorption-addiction model*, people lacking a clear sense of self become absorbed with celebrities to attain a more complete identity. This parasocial relationship strengthens over time, as individuals become addicted to their absorption and seek greater and more personal links [73]. According to the *empty-self model*, autonomous persons who value self-containment and self-sufficiency often have to sacrifice interpersonal relationships. These individuals, referred to as ‘empty selves’, have a consistent emotional need that celebrity attachment can fulfill [73]. Therefore, both traits of celebrities and their fans foster strong parasocial relationships, which enhance the former’s influence. Particularly in people suffering from poor mental health, attachments can progress to a borderline-pathological level of celebrity worship, in which parasocial relationships irrationally substitute for real life [75].

### Social networks (sociology)

The widespread uptake of celebrity medical advice can also be explained as a social contagion that diffuses through social networks. *Social networks* are systems of people linked through personal connections, such as family, marital and friendship ties [76]. Observational studies have found these interconnections to have significant effects on people’s health, including smoking [77], obesity [78], sexual activities [79] and happiness [80]. One person’s health decisions create externalities, by which connected individuals experience indirect consequences [81]. Within this interrelated system, clusters of people sharing common health behaviors form. Although celebrities only have loose social ties to most people, their newsworthiness and star quality, and the intense parasocial relationships some individuals have allow them to feature prominently within social networks. Thus, celebrities have great influence as medical advisors: as with Angelina Jolie’s announcement of her double mastectomy, celebrities’ messages reach many people simultaneously, and diffuse across social ties to affect diverse clusters. As social networks broaden with the development of social media technologies [82], celebrities can reach broader audiences faster and more intensely than ever before [83]. Indeed, online networks have become a key tool for celebrities such as Gwyneth Paltrow [84] and Jessica Alba [85] to disseminate medical advice.

### Commodification and social capital (sociology)

The ways in which people look to celebrities for health advice may stem from the broader context of consumerism. In our current capitalist society, celebrity culture appears to be one of the many entities that have been *commoditized*[86]. Celebrities themselves have become products that can be bought and sold. As old celebrities fade into obscurity and breakout stars gain newfound fame, there is a consistently refreshed stock of articles for trade [87]. But celebrities are not just inanimate objects for sale: consumers also “purchase” celebrities by acquiring their endorsed products, mimicking their lifestyles, and heeding their medical advice.

In sociology, these parasocial relationships have been conceptualized as a means of acquiring celebrities’ *social capital:* the benefits and resources accrued through social relationships [88]. Although acquiring the same coveted status of famous people is nearly impossible, doing what celebrities do and imitating their behaviors is a strategy for people seeking to raise their social status [86]. Celebrities, in this sense, have become resources in forming consumers’ social identities, used to shape the ways people see themselves and want others to see them [86]. Following celebrity medical advice may be a method for consumers to gain social capital and participate in the practices that make celebrities ‘special’, thereby elevating them to the upper echelons of society.

### Social construction of reality (sociology)

The ways in which people evaluate, interpret and perceive health information can also be influenced by celebrities. According to *social constructivism*, reality is a cultural product, formed by people’s interactions with each other and their environments [89]. The ways individuals create and learn knowledge is determined by the social activities in which they engage. This applies both to the social construction of health information as well as the social reconstruction of this information by each and every person in different ways depending on their unique social environment. This mechanism of celebrity influence is supported by a recent interview-based study’s finding that people assess information about vaccination differently: *acceptors* wholly accept social norms, *reliers* follow the norms of their social networks, and *searchers* independently seek whatever information they need [90]. Celebrities modify the ways all three types of people evaluate, interpret and perceive health information and consequently influence their health behaviors, albeit in different ways.

For acceptors, celebrities shape social norms that are internalized and acted upon. Much of the interest in detox cleansing and fasting, for example, can be attributed to celebrities like Salma Hayek and Ashton Kutcher who have made such behaviors socially acceptable and popular for weight loss and reducing gastrointestinal malaises. For reliers, many of their friends, family members and colleagues may follow or discuss celebrity medical advice, which indirectly encourages them to act similarly. For searchers, the information they gather may knowingly or unknowingly include advice from celebrities, especially as the internet burgeons with the health information they share. This means that all types of people, not just gossipmongers or people with low self-esteem, can be affected by the ways celebrities shape the social construction and reconstruction of health information.

## Discussion

There are strong biological, psychological and social explanations for why people adore celebrities and trust their medical advice (see Summary of key findings). Rather than being a product of health illiteracy, the inclination to mirror a celebrity’s health behaviors may stem from very particular processes, such as a desire to acquire the traits that make celebrities special, the activation of neural pathways involved in enhancing trust, or the spread of behaviors throughout social networks. Given the considerable influence that celebrities have, their public health engagements can thus act as either an aid or threat to health. Their influence can be harnessed to disseminate advice based on the best-available research evidence, but it can just as easily be abused to promote useless products and bogus treatments.

### Summary of key findings

-  Celebrities can strongly influence people’s health-related behaviors, which can either be beneficial or harmful depending on the accuracy of their advice.
-  Powerful biological, psychological and social forces contribute to celebrities’ influence, making it a serious phenomenon worthy of serious address.
-  Health professionals are encouraged to discuss the merits and faults of celebrity medical advice with patients and ensure that patients know how to access and assess credible health information.
-  Regulations could help contain the most-damaging celebrity health endorsements or require celebrities to reveal conflicts of interest.
-  Governments, medical associations, research groups and healthcare organizations can harness the influence of celebrities by working with them to disseminate evidence-based advice and promote health literacy.

Health professionals and public health practitioners must therefore work to ensure that the public acts upon sound medical advice. Health professionals, who certainly should have the most medical credibility, can counter celebrities’ influence by speaking to their patients about the merits and faults of celebrity advice, where they can find accurate health information and the trustworthiness of different sources. The times when patients mention the latest celebrity endorsement, fad diet or miracle cure should not be seen as annoyances. Quickly dismissing celebrity-recommended remedies can weaken the doctor-patient relationship, particularly in cases of strong celebrity adoration. Instead, they should be seen as meaningful opportunities to start important educational conversations. Doing so will not only inform patients on the kinds of health behaviors that are truly beneficial, but also encourage patients to place more trust in their physicians. In addition, there is too often a disconnect between what the evidence says and what is reported by the media: for example, 68% of articles reporting on Angelina Jolie’s preventive double mastectomy did not discuss the rarity of BRCA mutations [91]. Doctors and researchers should thus work with media outlets to ensure accurate and actionable information is disseminated to the public.

The medical community can also improve its efforts to increase public understanding of health issues and discredit the most egregious examples of celebrity advice. One method may be to enact restrictions on celebrity endorsements, much like how advertisements for medicines are regulated, to ensure the promoted messages are supported by research evidence. Requiring celebrities to disclose any conflicts of interest, such as partnerships with pharmaceutical companies or the amount of financial compensation received, may also be an effective option. A potentially powerful strategy may be to actually work with celebrities. If governments, research groups and health professional associations can leverage the clout of celebrities – partnering with them in productive ways to disseminate the best available research evidence and share basic critical appraisal skills – celebrities can be used as a powerful tool for health literacy and health promotion. Public health authorities can take inspiration from previous partnerships that have mobilized celebrity influence for good. British chef Jamie Oliver collaborated with government officials and charities to make school meals healthier in the United Kingdom, an effort that was found to have had a lasting effect on students’ educational performance [92]. Actor Glenn Close is a recognized advocate for mental illness [93], and in 2011 comedian Stephen Fry became the president of Mind, an organization that supports individuals in the United Kingdom living with mental health problems [94]. Model Christy Turlington released a commercial with the Centers for Disease Control and Prevention urging viewers to refrain from smoking [95]. Collaborations with celebrities can be further complemented by counter-marketing and social media efforts to discredit incorrect celebrity messages while spreading evidence-based advice.

This meta-narrative analysis has several methodological strengths. First, it represents the greatest effort so far to synthesize research from across disciplines on celebrity influence. Second, the literature review was conducted as systematically as possible – limiting bias – without sacrificing the breadth of studies covered. There are also at least a few methodological weaknesses. First, by taking a broad interdisciplinary approach to the analysis, the nuances of individual studies were invariably lost. Second, despite the systematic search strategy, we cannot be confident that *all* relevant studies were captured due to the expansive nature of the literature and potential differences between databases.

The cumulative evidence presented in this meta-narrative analysis indicates that mechanisms of celebrity influence have been widely studied in multiple disciplines. Nevertheless, additional research within and across disciplines that critically analyzes these mechanisms is an important future step to ensure celebrities do more good for public health than harm. Although comparisons between disciplines are beyond the scope of this exploratory review, we see that the marketing and psychology disciplines have studied the phenomenon most extensively, while the neuroscience and economics literatures have focused relatively less on this matter. Future interdisciplinary collaborations would be helpful in identifying the most important mechanisms of celebrity influence, understanding the full benefits, costs, risks of harms and trade-offs of celebrities acting as medical advisors, and finding the best ways to address this challenge.

## Conclusion

Ultimately, we need to fundamentally rethink and better understand where people obtain their health information and what makes them act upon it. Understanding why people follow celebrities’ medical advice and developing strategies to exploit the implicated biological, psychological and social processes to promote evidence-based practices represent a good start. Doing so may foster constructive relationships with celebrities, allowing them to become important partners in improving public health.

## Electronic supplementary material

Additional file 1: **Systematic Literature Search Results includes the detailed search results used to inform this study.** (DOCX 94 KB)
