# Supplementary material for: Biological, psychological and social processes that explain celebrities’ influence on patients’ health-related behaviors
Source: Arch Public Health. 2015 Jan 19;73(1):3. doi: 10.1186/2049-3258-73-3 (PMC4429495; doi:10.1186/2049-3258-73-3)
Supplement: Supplementary file 1 — Additional file 1: Systematic Literature Search Results includes the detailed search results used to inform this study. (DOCX 94 KB) [file 13690_2014_5074_MOESM1_ESM.docx]

**Additional file 1 Web Appendix 1: Systematic Literature Search Results**

**Search Results**

**Medical Database**

***PubMed, 1966-2013 (447 identified, 14 relevant)***

1. Boyland EJ, Harrold JA, Dovey TM, Allison M, Dobson S, Jacobs MC, Halford JC. Food choice and overconsumption: effect of a premium sports celebrity endorser. J Pediatr. 2013 Mar 9. doi:10.1016/j.jpeds.2013.01.059. [Epub ahead of print]

OBJECTIVE: To determine whether exposure to celebrity endorsement in television (TV) food advertising and a nonfood context would affect ad libitum intake of the endorsed product and a perceived alternative brand. STUDY DESIGN: A total of 181 children from the UK aged 8-11 years viewed 1 of the following embedded within a cartoon: (1) a commercial for Walker's Crisps (potato chips), featuring a long-standing celebrity endorser; (2) a commercial for a savory food; (3) TV footage of the same endorser in his well-known role as a TV presenter; or (4) a commercial for a nonfood item. Children's ad libitum intake of potato chips labeled "Walker's" and "supermarket brand" was measured using ANOVA. RESULTS: Children who viewed the endorsed commercial or the TV footage of the endorser outside of a food context consumed significantly more of the Walker's chips compared with children in other groups. These children did not reduce their intake of the supermarket brand product to compensate; thus, the endorser effect contributed to overconsumption. CONCLUSION: The influence of a celebrity endorser on food intake in children extends beyond his or her role in the specific endorsed food commercial, prompting increased consumption of the endorsed brand even when the endorser has been viewed in a nonfood context. Our data suggest that the ubiquitous nature of celebrity media presence may reinforce unhealthy eating practices in children, although research with other endorsers is needed.

1. Casey MK, Allen M, Emmers-Sommer T, Sahlstein E, Degooyer D, Winters A, Wagner AE, Dun T. When a celebrity contracts a disease: the example of Earvin "Magic" Johnson's announcement that he was HIV positive. J Health Commun. 2003 May-Jun;8(3):249-65.

This meta-analysis summarizes the available data concerning the impact that the public announcement that Earvin "Magic" Johnson, a National Basketball Association All-Star, had tested positive for HIV. The results demonstrate that the announcement increased the level of accurate knowledge in persons, the number of persons getting tested for HIV, and the desire to obtain more information about HIV and AIDS. For adults the impact of the announcement was to increase the perception of vulnerability while for children/adolescents the announcement diminished the perception of risk.

1. Chapman S. Does celebrity involvement in public health campaigns deliver long term benefit? Yes. BMJ. 2012 Sep 25;345;e6364. doi:10.1136/bmj.e6364.
2. Chapman S, Leask J-A. Paid celebrity endorsements in health promotion: a case study from Australia. Health Promot Int. 2001;16(4):333-8.

In late 1998, Australian cricketer Shane Warne was allegedly paid A$200 000 (£78 060, US$123 000) by a pharmaceutical company to publicize his attempt to stop smoking. Warne failed to stop, and his continuing smoking remained newsworthy more than a year later. The arrangement caused considerable media controversy about the ethics of payment for charitable or socially worthy actions. This paper explores the community's reaction to payment for modelling a healthy behaviour, discussing the values that Warne transgressed and whether these mattered, given that the campaign caused an unprecedented rise in the use of nicotine replacement therapy.

1. Cram P, Fendrick AM, Inadomi J, Cowen ME, Carpenter D, Vijan S. The impact of celebrity promotional campaign on the use of colon cancer screening: the Katie Couric effect. Arch Intern Med. 2003 Jul 14;163(13):1601-5.

BACKGROUND: Public participation in many preventive health programs is suboptimal. While various interventions to increase participation have been studied, the impact of a celebrity spokesperson on cancer screening has not been rigorously examined. The objective of this study was to assess the impact of Katie Couric's March 2000 Today Show colorectal cancer awareness campaign on colonoscopy rates. METHODS: A population-based observational study was conducted using 2 different data sources: (1) The Clinical Outcomes Research Initiative (CORI) database-a voluntary consortium of 400 endoscopists who performed 95 000 colonoscopies from July 1998 to December 2000; and (2) 44 000 adult members of a managed care organization. Using change point analyses and linear regression models, we compared colonoscopy utilization rates before and after Ms Couric's March 2000 television series. RESULTS: The number of colonoscopies performed per CORI physician per month after Ms Couric's campaign increased significantly (15.0 per month before campaign; 18.1 after campaign; P<.001). After adjusting for temporal trends, a significantly higher postcampaign colonoscopy rate was sustained for 9 months. Analysis also demonstrated a trend toward an increase in the percentage of colonoscopies performed on women (43.4% before campaign; 47.4% after campaign; P =.054). Colonoscopy rates also increased significantly in the managed care organization after Ms Couric's campaign (1.3 per 1000 members per month before; 1.8 after; P<.001). CONCLUSIONS: Katie Couric's televised colon cancer awareness campaign was temporally associated with an increase in colonoscopy use in 2 different data sets. These findings suggest that a celebrity spokesperson can have a substantial impact on public participation in preventive care programs.

1. Distefan JM, Pierce JP, Gilpin EA. Do favorite movie stars influence adolescent smoking initiation? Am J Public Health. 2004 Jul;94(7):1239-44.

OBJECTIVES: We sought to determine whether adolescents whose favorite movie stars smoke on-screen are at increased risk of tobacco use. METHODS: During interviews, adolescent never smokers taking part in the California Tobacco Survey nominated their favorite stars. We reviewed popular films released during 1994 through 1996 to determine whether stars smoked on-screen in at least 2 films. RESULTS: One third of never smokers nominated a star who smoked on-screen, which independently predicted later smoking risk (odds ratio [OR] = 1.36; 95% confidence interval [CI] = 1.02, 1.82). The effect was strong among girls (OR = 1.86; 95% CI = 1.26, 2.73). Among boys, there was no independent effect after control for receptivity to tobacco industry promotions. CONCLUSIONS: Public health efforts to reduce adolescent smoking must confront smoking in films as a tobacco marketing strategy.

1. Dixon H, Scully M, Wakefield M, Kelly B, Chapman K, Donovan R. Parent's responses to nutrient claims and sports celebrity endorsements on energy-dense and nutrient-poor foods: an experimental study. Public Health Nutr. 2011 Jun;14(6):1071-9. doi:10.1017/S1368980010003691.

OBJECTIVE: To assess parents' responses to common, potentially misleading strategies for marketing energy-dense and nutrient-poor (EDNP) child-oriented foods. DESIGN: Between-subjects online experiment to test whether nutrient claims and sports celebrity endorsements on the front of packs of EDNP products lead parents to prefer and rate these foods more favourably. SETTING: Australia. SUBJECTS: A total of 1551 parents of children aged 5-12 years, who were the main household grocery buyers. RESULTS: Inclusion of nutrient claims or sports celebrity endorsements on EDNP products led parents to perceive these products to be more nutritious than if they did not include such promotions. When asked to choose between a pair of different products (EDNP v. healthier), 56 % of parents did not read a nutrition information panel (NIP) before making their choice and this did not differ by promotion condition. These parents were more likely to choose an EDNP product if it included a nutrient claim (OR = 1.83, 95 % CI 1.31, 2.56; P < 0.001) or sports celebrity endorsement (OR = 2.37, 95 % CI 1.70, 3.32; P < 0.001). Sports celebrity endorsements also enhanced parent's perceptions of typical consumers of the product, perceptions of product healthiness and quality, as well as purchase intentions. CONCLUSIONS: Nutrient claims and sports celebrity endorsements tip consumer preferences towards EDNP products bearing such promotions, especially among the majority who do not read the NIP. As parents largely determine what foods are available to children at home, it is critical that initiatives aimed at reducing the persuasive impact of food marketing include this target group.

1. Holmes D. Celebrities and cancer campaigns: time for a rethink? Lancet Oncol. 2010 Apr;11(4):320.
2. Larson RJ, Woloshin S, Schwartz LM, Welch HG. Celebrity endorsements of cancer screening. J Natl Cancer Inst. 2005 May 4;97(9)693-5.

Celebrities often promote cancer screening by relating personal anecdotes about their own diagnosis or that of a loved one. We used data obtained from a random-digit dialing survey conducted in the United States from December 2001 through July 2002 to examine the extent to which adults of screening age without a history of cancer had seen or heard or been influenced by celebrity endorsements of screening mammography, prostate-specific antigen (PSA) testing, or sigmoidoscopy or colonoscopy. The survey response rate was 72% among those known to be eligible and 51% among potentially eligible people accounting for those who could not be contacted. A total of 360 women aged 40 years or older and 140 men aged 50 years or older participated in the survey. Most respondents reported they "had seen or heard a celebrity talk about" mammography (73% of women aged 40 years or older), PSA testing (63% of men aged 50 years or older), or sigmoidoscopy or colonoscopy (52% of adults aged 50 years or older). At least one-fourth of respondents who had seen or heard a celebrity endorsement said that the endorsement made them more likely to undergo mammography (25%), PSA testing (31%), or sigmoidoscopy or colonoscopy (37%).

1. Moynihan R. The intangible magic of celebrity marketing. PLoS Med. 2004 Nov;1(2):e42.
2. Peterson M. Heartfelt advice, hefty fees: companies pay stars to mention prescription drugs [Internet]. New York (NY): New York Times; 2002 Aug 11 [cited 2013 Jan 19]. Available from: http://www.nytimes.com/2002/08/11/business/heartfelt-advice-hefty-fees.html?pagewanted=all&src=pm
3. Rayner G. Does celebrity involvement in public health campaigns deliver long term benefit? No. BMJ. 2012 Sep 25;345;e6362. doi:10.1136/bmj.e6362.
4. Tanne JH. Celebrity illnesses raise awareness but can give wrong message. BMJ. 2000 Nov 4;321(7269):1099.
5. Tanne JH. Does publicity about celebrity illness improve public health? West J Med. 2001 Feb;174(2):94-5.

**Marketing/Business Database**

***Business Source Complete, 1886-2013 (461 identified, 14 relevant and unique)***

1. Casais B, Proença JF. Inhibitions and implications associated with celebrity participation in health-related social marketing: an exploratory research focused on HIV prevention in Portugal. Health Mark Q. 2012 Jul-Sep;29(3):206-22. doi:10.1080/07359683.2012.705642.

This article discusses motivations and inhibitions among celebrities to participate in health-related social marketing. The research identifies the implications that this involvement may have upon their lives. Results from in-depth interviews with 27 Portuguese celebrities show that they expect a fee for endorsements of commercial and government social marketing, despite the positive image they may gain from endorsing public health. The results demonstrate an absence of celebrity prejudice against HIV because of its serious nature and the social stigma attached to AIDS. This research suggests there is a positive bias and presents helpful information for negotiations between institutions and celebrities.

1. Cronley ML, Kardes FR, Goddard P, Houghton DC. Endorsing products for the money: the role of the correspondence bias in celebrity advertising. Adv Consum Res. 1999;26(1):627-31.

Although there have been many studies regarding celebrity endorsed advertising, little work has been done examining why celebrity advertising is effective. The present study examines celebrity advertising in terms of the correspondence bias. This bias is the tendency to assume that a person's behavior is a reflection of their true underlying dispositions when in fact, their behavior could be explained by situational factors. Results show that people exhibit correspondent inferences when evaluating celebrity endorsed ads, despite knowing the celebrity was paid a large endorsement fee. These inferences are subsequently related to brand evaluations.

1. Erdogan BZ. Celebrity endorsement: a literature review. J Market Manag. 1999 May;15(4):291-314. doi:10.1362/026725799784870379.

Use of celebrities as part of marketing communications strategy is a fairly common practice for major firms in supporting corporate or brand imagery. Firms invest significant monies in juxtaposing brands and organisations with endorser qualities such as attractiveness, likeability, and trustworthiness. They trust that these qualities operate in a transferable way, and, will generate desirable campaign outcomes. But, at times, celebrity qualities may be inappropriate, irrelevant, and undesirable. Thus, a major question is: how can companies select and retain the 'right' celebrity among many competing alternatives, and, simultaneously manage this resource, while avoiding potential pitfalls? This paper seeks to explore variables, which may be considered in any celebrity selection process by drawing together strands from various literature.

1. Gurel-Atay E, Kahle L. Celebrity endorsements and advertising effectiveness: the importance of value congruence. Adv Consum Res. 2010;37:807-9.

The article focuses on the study that investigates the impact of celebrity endorsements on advertising effectiveness. The study shows that even a single advertisement based on value congruence is capable of affecting attitude toward brand and purchase intention. It provides some insights that value congruence adds to the variance explained in advertising effectiveness, beyond and above attractiveness and expertise dimensions of the match-up hypothesis.

1. Kerrigan F, Browlie D, Hewer P, Daza-LeTouze C. ‘Spinning’ Warhol: celebrity brand theoretics and the logic of the celebrity brand. J Market Manag. 2011 Dec;27(13/14):1504-24. doi:10.1080/0267257X.2011.624536.

The paper takes as its subject celebrity and consumption and the cultural logic of the celebrity brand. It introduces the concept of celebritisation as the engine of celebrity culture, discussing ways in which celebrity brands operate as ‘map-making’ devices which situate consumers within networks of symbolic resources. We construct particulars via an investigative narrative that draws critically upon published accounts of the life and work of Andy Warhol, generating observations of signature practices and technologies of formation of Celebrity Brandhood. Within an inductive architecture we modulate to celebrity brands as transmediated marketing accomplishments which trade upon allure, glamour and charisma, constructed around rituals of transition, belonging, intimacy, and affect. We suggest that at the heart of the machinery of the cultural logic of the Celebrity Brand is the mediated spectacle as a field of social invention and transformation. In this way, the paper opens up pathways toward further interpretive analyses of celebrity brands, articulating the basis of accounts of Celebrity Brand Theoretics.

1. Maceo B. Think big. Mark Health Serv. 2011 Winter;31(1):26-31.

The article focuses on the marketing strategy of City of Hope, one of the largest providers of cancer care in California, to build national brand awareness. It says that the organization has integrated national awareness goals into its institutional strategic planning process on a local and nonprofit budget. It states that the cause-related marketing team of the organization has added business development to its role and broadened its marketing outreach and relationships with celebrities. Moreover, the web team has moved into social media and marketing analytics to attract younger national audience. Furthermore, the entire marketing team has collaborated with its fundraisers and philanthropic efforts through its Music and Entertainment Industry, board connections and patient relationships.

1. Morin DT, Ivory JD, Tubbs M. Celebrity and politics: effects of endorser credibility and sex on voter attitudes, perceptions, and behaviors. Soc Sci J. 2012 Dec;49(4):413-20. doi:10.1016/j.soscij.2012.09.011.

While much research examines the effects of celebrity endorsements in commercial advertising, scholars have only recently sought to investigate the effects of celebrity endorsements of politicians on voter perceptions and behavior. This study expands existing research on celebrity political endorsement effects via an experiment exploring effects of different versions of a news story describing a celebrity’s endorsement of a political candidate on participants’ voting attitudes, perceptions of candidate credibility, and voting behavioral intent. Although participants perceive credibility differences between high- and low-credibility celebrities, neither endorser credibility nor endorser sex impact attitudes toward the endorsed candidate, perceptions of the candidate’s credibility, or intended voting behavior. Conceptual relationships to other studies on celebrity endorsement effects are discussed, as are implications, limitations, and directions for future research.

1. Ohanian R. The impact of celebrity spokespersons’ perceived image on consumers’ intention to purchase. J Advertising Res. 1991 Feb/Mar;31(1):46-54.

The article discusses research questions dealing with the use of celebrity spokespeople in advertising. The reason behind the popularity of celebrity advertising is the advertisers' belief that messages delivered by well-known personalities achieve a high degree of attention and recall for some consumers. While the idea is intuitively appealing, it is strengthened by an appropriate connection between the celebrity and the product endorsed or by the celebrity's personification of some aspect of the product. The research on source credibility has shown that in most situations a highly credible source is more effective than a less credible source. Through the increasing use of celebrities as endorsers of products, services, and social causes, attractiveness has become an important dimension of source credibility. To discern the importance of physical attractiveness, one has only to watch television or to look at print advertisements. Most television and print advertisements use physically attractive people. Consumers tend to form positive stereotypes about such people, and, in addition, research has shown that physically attractive communicators are more successful in changing beliefs than are unattractive communicators.

1. Patra S, Datta SK. Celebrity selection & role of celebrities in creating brand awareness and brand preference – a literature review. J Market Comm. 2012 Sep-Dec;8(2):48-57.

The phenomenon of celebrity endorsement has attracted a lot of attention from researchers across the globe. It has been studied from different perspective in different cultural context. In the Indian context, this phenomenon started gaining prominence with the opening of our economy in the early 1990's. The Hindi film stars from Bollywood and the Indian cricketers are the most prominent celebrities and are in great demand among advertiser and their advertising agencies as brand endorsers. The primary focus in this paper is to present a comprehensive review of literature about the various models available for selecting a celebrity as well as about the role of celebrities in creating brand awareness and brand preference among the target audience. The paper also highlights the brand endorsement details of the 20 Indian celebrities selected for the study. The findings show that prominent models used for celebrity selection are the Source Credibility Model, Source Attractiveness Model, Meaning Transfer Model and "Q" rating score. It is interesting to see that Multiple Brand Endorsement is a common phenomenon among Indian celebrities across different product categories.

1. Pease A, Brewer PR. The Oprah factor: the effects of a celebrity endorsement in a presidential primary campaign. Int J Press/Polit. 2008 Oct;13(4):386-400. doi:10.1177/1940161208321948.

In 2007, popular daytime talk show host Oprah Winfrey endorsed Barack Obama, a candidate for the 2008 Democratic presidential nomination. This study uses data from an experiment to examine the impact of news about her endorsement. Exposure to such news did not influence the extent to which participants held favorable opinions toward Obama or the extent to which they saw him as likable. On the other hand, reading about the endorsement did lead participants to see Obama as more likely to win the nomination and to say that they would be more likely to vote for him. These findings suggest that research on celebrity endorsements should consider not only effects on candidate support but also subtler effects, such as those on viability assessments.

1. Rossiter JR, Smidts A. Print advertising: celebrity presenters. J Bus Res. 2012 Jun;65(6):874-9. doi:10.1016/j.jbusres.2011.01.010.

This study validates Rossiter and Percy’s (1987) hook theory of presenter characteristics, for celebrity presenters. Firstly, by employing a product-alone control group, the study demonstrates that some celebrity-product pairings have a good fit and can persuade whereas others have no effect or represent such an obviously poor fit that they dissuade consumers from buying the product. Secondly, the study suggests that good fit, and thus persuasion, for celebrity presenters, depends on the audience immediately perceiving that the celebrity is an expert user of the product (for all products) and is a positive role model (for high-risk products). On the other hand, the study reveals that failure of any of four of the celebrity's characteristics causes dissuasion by celebrity presenters; these failures include lack of high visibility (i.e., not widely well-known), perceived inexpertness as a user of the product (a strong negative hook that is probably the reason for the poor fit perception), lack of trust (though this is a weak dissuasive factor for celebrities) and, paradoxically, the celebrity being too likable (for low-risk products). Thirdly, the hook(s) conceptualization of presenter characteristics is superior to the conventional linear conceptualization in that a hook-scored regression model accounted for the same amount of variance in persuasion–dissuasion as did the linear model (adjusted R 2s of 41% vs. 43%) despite the hook model's handicap of at least one-third lower possible R 2 due to trichotomization of 7-point ratings into positive, neutral, and negative hooks.

1. Samman E, McAuliffe E, MacLachlan M. The role of celebrity in endorsing poverty reduction through international aid. International Journal of Nonprofit & Voluntary Sector Marketing. 2009 May;14(2):137-48. doi:10.1002/nvsm.339.

Celebrity endorsement' is a strategy that is gathering increasing momentum in attempts to develop public awareness of the plight of the poor. Understanding of public perceptions is clearly important for international organizations seeking to make use of celebrity in furthering their causes. This paper reports the results of a preliminary survey conducted among 100 members of the Irish public to evaluate levels of awareness of celebrity involvement in international development work and the public's opinions about such involvement. The survey instrument was semi-structured with some open-ended questions. The focus was on respondents' ability to identify celebrities associated with such work, and to elicit their opinions on those celebrities' perceived aims, knowledge of international development, and influence upon the respondent. It also requested opinions of the value of celebrity involvement more generally. The results suggest that respondents are generally able to distinguish between celebrities and their various causes. Most found their involvement to be valuable in raising the profile of charities, though only a small number claimed to be personally influenced by such activity. The respondents were fairly cynical as to the motives of most celebrities, whose involvement they felt served their own aims -- namely publicity, -- first and foremost. Most respondents were more likely to be influenced by their perceptions of the character of the celebrity rather than their causes. They respected celebrities they felt were genuinely committed to the causes they espoused, but paradoxically, they felt such commitment was best demonstrated by the celebrity keeping a low profile and not actively seeking publicity. Long-term commitment to a given cause was also highly regarded. The results are discussed with regard to theories of social persuasion and the dilemma's facing celebrities who get involve in endorsement of charity aid or campaigns. More research is necessary to substantiate and further develop our findings.

1. Sanbonmatsu DM, Mazur D, Pfeiffer BE, Kardes FR, Posavac SS. The less the public knows the better? The effects of increased knowledge on celebrity evaluations. Basic Appl Soc Psych. 2012 Nov/Dec;34(6):499-507. doi:10.1080/01973533.2012.728408.

Celebrities are figures that people like a lot but know little about. Two experiments investigated how celebrity evaluations are affected by increased knowledge. In Experiment 1, heightened knowledge of the political orientation, faith, and social attitudes of two prominent actors led to less favorable evaluations and greater differentiation in the evaluations of the actors along political and gender lines. In Experiment 2, increasing participants' cognizance of their limited knowledge of popular entertainers led to less positive evaluations and diminished credibility of the celebrities as spokespersons. The findings suggest that increasing knowledge and meta-knowledge of celebrities may diminish their marketability.

1. Till BD, Busler M. Matching products with endorsers: attractiveness versus expertise. J Consum Market. 1998;15(6):576-86. doi:10.1108/07363769810241445.

The article presents a study that examined the effectiveness of using endorsers for marketing products. This article has significant implications regarding the choice of appropriate celebrities for product endorsements. Understanding the effectiveness of endorsers is an important issue for both practitioners and academics. Indeed, there have been a number of studies that have examined whether, and under what conditions, celebrities make appropriate endorsers for products. Most of the empirical work on the match-up hypothesis has focused on the physical attractiveness of the endorser. The argument is that attractive celebrities are more effective endorsers for products which are used to enhance one's attractiveness. Early research found that the effectiveness of endorsers varies by product. Although the match-up hypothesis has generally been framed within the context of physical attractiveness, the purpose of this article is to explore whether it is attractiveness or expertise which makes for a stronger match-up factor.

**Communications Database**

***Communication and Mass Media Complete, 1915-2013 (213 retrieved, 14 relevant and unique)***

1. Amos C, Holmes G, Strutton D. Exploring the relationship between celebrity endorser effects and advertising effectiveness. Int J Advert. 2008;27(2):209-34.

This study provides a quantitative summary of the relationship between celebrity endorser source effects and effectiveness in advertising. The Kruskal-Wallis non-parametric test is used to identify the most influential celebrity endorser source effects on effectiveness. The role of celebrity/product fit, interaction effects, sample type, study setting, and country of study are also included as moderators. Results suggest negative celebrity information can be extremely detrimental to an advertising campaign. The source credibility model composed of celebrity trustworthiness, celebrity expertise, and celebrity attractiveness appears to capture the three most influential source effects on purchase intentions, brand attitudes and attitudes towards the advertisement.

1. Austin EW, Vord RV, Pinkleton BE, Epstein E. Celebrity endorsements and their potential to motivate young voters. Mass Commun Soc. 2008 Fall;11(4):420-36. doi:10.1080/15205430701866600.

Although scholars have long indicated concern regarding disaffected young voters, the 2004 presidential election tallied record turnout among this age group. This study explored how and why celebrity-endorsed, get-out-the-vote campaigns may have helped to persuade young voters aged 18 to 24 to participate in an election campaign by examining campaign influence on individual decision-making factors. During the fall semester of 2004, a convenience sample of 305 college students from introductory general education classes completed surveys assessing their political efficacy, involvement, complacency, and apathy. Findings indicated that receptivity to celebrity spokespeople predicted lower levels of complacency and higher levels of self-efficacy. Complacency had independent effects on involvement and self-efficacy. The results therefore suggest that these campaigns can potentially influence positive change in political engagement of the younger citizenry.

1. Choi SM, Rifon N. It is a match: the impact of congruence between celebrity image and consumer ideal self on endorsement effectiveness. Psychol Market. 2012 Sep;29(9):639-50. doi:10.1002/mar.20550.

Using celebrities for promoting products is a popular advertising strategy. The selection of celebrity endorsers is of great concern to advertisers given the large sums of money to secure their participation. To date, most academic research on celebrity endorser effectiveness has focused on endorser characteristics (e.g., source credibility) or a match between a product and the endorser (e.g., match-up hypothesis). The study presented here introduces a new dimension for understanding celebrity endorser effects, the congruence between a consumer's perception of a celebrity's personality characteristics with the consumer's self-concept. Consumers' self-concept is an important influence on purchase decisions (Ericksen, 1997; Graeff, 1996; Sirgy, 1982, 1985), and advertising has been viewed as the most effective tool for creating product images in relation to such self-concept (Sirgy, 1982). Drawn from the two streams of literature, this study proposes and tests an integrative model of celebrity endorsement by examining congruence effects of consumer self-concept and celebrity as well as product and celebrity. Results suggest that ideal congruity (congruence between consumers' ideal self-image and celebrity image) adds explanatory power to a congruence model of celebrity endorser effects. Implications for advertisers and suggestions for future research are discussed.

1. Elberse A, Verleun J. The economic value of celebrity endorsements. J Advertising Res. 2012 Jun;52(2):149-65. doi:10.2501/JAR-52-2-149-165.

What is the pay-off to enlisting celebrity endorsers? Although effects on stock returns are relatively well documented, little is known about any impact on sales-arguably a metric of more direct importance to advertising practitioners. This study of athlete endorsements finds there is a positive pay-off to a firm's decision to sign an endorser, and that endorsements are associated with increasing sales in an absolute sense and relative to competing brands. Furthermore, sales and stock returns jump noticeably with each major achievement by the athlete. However, whereas stock-return effects are relatively constant, sales effects exhibit decreasing returns over time. Implications for practitioners are outlined.

1. Fleck N, Korchia M, Le Roy I. Celebrities in advertising: looking for congruence or likability? Psychol Market. 2012 Sep;29(9):651-62. doi:10.1002/mar.20551.

The choice of a celebrity endorser for a brand is an important topic in advertising and marketing, as considerable time and effort resources are dedicated to finding the right celebrity to represent a given organization. Celebrities used as endorsers in advertisements are often very popular ones. However, from a cognitive point of view (and a more academic one), congruence between brand and celebrity seems to be very important too. Based on affective and cognitive theories to explain endorsement effectiveness, congruence between brand and celebrity is shown to be as effective as celebrity likability. Moreover, congruence between brand and celebrity as well as celebrity likability have an impact on the predisposition toward the ad, which in turn influences brand beliefs and purchase intention.

1. Goldsmith RE, Lafferty BA, Newell SJ. The impact of corporate credibility and celebrity credibility on consumer reaction to advertisements and brands. J Advertising. 2000 Fall;29(3):43-54.

Advertisers frequently use endorsers or spokespersons as credible sources to influence consumers' attitudes and purchase intentions. Corporate credibility--the reputation of a company for honesty and expertise--is another type of source credibility that can influence consumer reactions to ads and shape brand attitudes. The present study assessed the impact of endorser and corporate credibility on attitude-toward-the-ad, attitude-toward-the-brand, and purchase intentions. We surveyed 152 adult consumers who viewed a fictitious ad for Mobil Oil company. They rated the credibility of the ad's endorser, the credibility of the company, and attitude-toward-the-ad, attitude-toward-the-brand, and purchase intentions. Path analysis confirmed that endorser credibility had its strongest impact on Aad while corporate credibility had its strongest impact on AB. The findings suggest that corporate credibility plays an important role in consumers' reactions to advertisements and brands, independent of the equally important role of endorser credibility.

1. Jackson DJ. Selling politics: the impact of celebrities’ political beliefs on young Americans. J Polit Market. 2007;6(4):67-83. doi:10.1300/J199v06n04_04.

Parents, the press and political scientists have increasingly shown interest in the influence of the entertainment media on young adults' political beliefs. This research examines the impact of celebrities' endorsements of certain political beliefs on the attitudes on young Americans, and finds that young people are significantly more likely to agree with a position when it is endorsed by a celebrity. Causes and candidates are cautioned to select celebrity endorsers carefully, because not all celebrities are created equal when it comes to influencing public opinion, with the evidence suggesting celebrity status in and of itself may not be most important factor, but credibility and appropriate match-up between idea and endorser matter as well.

1. Jackson DJ, Darrow TIA. The influence of celebrity endorsements on young adults’ political opinions. Harv Int J Press/Pol. 2005 Summer;10(3):80-98. doi:10.1177/1081180X05279278.

This research contributes to our understanding of the increasing mixture of entertainment and politics by examining the impact of the political statements made by celebrities on the opinions of Anglophone Canadian youth. A recent survey of young Canadians enrolled in first-year university political science courses indicates young people's level of agreement with certain political statements is increased by the endorsement of these positions by Canadian celebrities from the realms of popular music and sports. These results suggest that celebrity endorsements make unpopular statements more palatable, while increasing the level of agreement with already popular opinions.

1. Keel A, Nataraajan R. Celebrity endorsements and beyond: new avenues for celebrity branding. Psychol Market. 2012 Sep;29(9):690-703. doi:10.1002/mar.20555.

The authors assess the extant research in the area of celebrity endorsement and point out the need for continuing research in celebrity marketing. Suggestions for future research are made in a wide breadth of areas, spanning from celebrity endorsements to novel areas of celebrity branding. The authors propose three celebrity-branding strategies and discuss the factors that may influence their effectiveness. Other areas for future research in celebrity marketing that are discussed include ethical marketing to vulnerable consumers and social marketing. The authors conclude with suggestions regarding theories and methods that can be used for future research in celebrity marketing.

1. Lee J, Thorson E. The impact of celebrity-product incongruence on the effectiveness of product endorsement. J Advertising Res. 2008 Sep;48(3):433-49. doi:10.2501/S0021849908080446.

The present study examines how different degrees of celebrity--product incongruence influence the persuasiveness of celebrity endorsement. Schema-congruity framework provides the theoretical basis for suggesting that a moderate mismatch between a celebrity's image and a product's image would produce more favorable responses to advertisements than either a complete match or an extreme mismatch. This study also looks at how consumer characteristics, namely an individual's own levels of enduring involvement with a product category, moderate schema congruity effects. Two experiments were conducted to test these issues using two types of match-up factors: physical attractiveness and expertise of a celebrity endorser. The results show that celebrity endorsements are evaluated more favorably in terms of purchase intention when there is a moderate mismatch than when there is either a complete match or an extreme mismatch. Such effects are found to be more pronounced among participants with higher product involvement than those with lower product involvement.

1. Lord KR, Putrevu S. Informational and transformational responses to celebrity endorsements. Journal of Current Issues and Research in Advertising. 2009 Spring;31(1):1-13. doi:10.1080/10641734.2009.10505253.

Using three studies, this research examines the relationship between dimensions of celebrity endorser credibility (attractiveness, expertise, trustworthiness) and consumer motivation (informational and transformational). Studies 1 and 2 reveal celebrity expertise and trustworthiness are the primary determinants of informational processing, while attractiveness is the principal variable driving transformational processing. The third study finds that teenage consumers, though affected by informational and transformational motivations, are less likely than adults to discriminate between attractiveness, trustworthiness, and expertise, forming their judgments instead on the basis of a unidimensional perception of celebrity credibility.

1. Premeaux SR. The attitudes of the middle class male and female consumers regarding the effectiveness of celebrity endorsers. J Promot Manag. 2005;11(4):33-48. doi:10.1300/J057v11n04_04.

This study analyzes consumer perceptions regarding the effectiveness of celebrity endorsers in relation to the AIDA framework and the match-up hypothesis. Findings indicate that celebrity endorsers influence both men and women, but men were influenced to a greater degree than women. The main AIDA influence is the celebrity endorser's ability to get and hold attention. Celebrity endorsers also appear to help products stand out from the competition, and make ads more memorable, as well as enhancing brand awareness, particularly for desired products. Indications are that the most important celebrity source characteristic is expertise, which apparently can overcome certain celebrity character flaws, such as the lack of trustworthiness and likeability.

1. Silvera DH, Austad B. Factors predicting the effectiveness of celebrity endorsement advertisements. Eur J Marketing. 2004;38(11/12),1509-26. doi:10.1108/03090560410560218.

This research examines whether consumers infer that celebrity endorsers like the products they endorse, and presents a model using these inferences and other characteristics of the endorser to predict attitudes toward the endorsed product. Participant in two experiments examined written endorsement advertisements and were asked to infer the extent to which the endorser truly liked the advertised product and to rate the endorser's attractiveness, similarity to themselves, and knowledge of the product. Attitudes toward the advertisement, the endorser and the product were also measured. The resulting model indicated that product attitudes were predicted by inferences about the endorser's liking for the product and by attitudes toward the endorser.

1. Thomson M. Human brands: investigating antecedents to consumers’ strong attachments to celebrities. J Marketing. 2006 Jul;70{3}:104-19.

This article explores recent advances in self-determination research to address why consumers develop strong attachments to "human brands," a term that refers to any well-known persona who is the subject of marketing communications efforts. Study 1 uses a survey that is analyzed with structural equation modeling. Study 2 is qualitative and offers corroborating evidence for the proposed theoretical model. Study 3 extends the model with a more naturalistic sample and tests several alternative hypotheses using hierarchical regression. The results suggest that when a human brand enhances a person's feelings of autonomy and relatedness and does not suppress feelings of competence, the person is likely to become more strongly attached to it. This article documents that strong attachments are predictive of satisfied, trusting, and committed relationships and proposes that attachment strength may be a parsimonious proxy for consumer-brand relationship strength. The results imply that benefits would accrue to organizations such as entertainment firms and political parties that establish direct and routine interaction between human brands and consumers, that human brands to which consumers are attached offer significant potential as endorsers, and that organizations should address how to make the human brands they manage more authentic.

**Psychology Database**

***PsycINFO*, 1806-2013 (333 retrieved, 19 relevant and unique)**

1. Basil MD. Identification as a mediator of celebrity effects. J Broadcast Electron. 1996;40(4):478-95. doi:10.1080/08838159609364370.

Tested the hypothesis that the effects of identification with a celebrity will mediate the adoption of attitudinal and behavioral positions advocated by that celebrity. The effects of identification with a celebrity were tested through young adults' identification with Earvin "Magic" Johnson (MJ), their AIDS-related concern, perceived risk, and sexual behavior. Data were collected from 147 students of the University of Hawaii at Manoa through a questionnaire about 1 yr after MJ's announcement that he contracted HIV. In examining MJ's effects on the respondents, a series of regressions was used. Overall results supported the stated hypothesis. This finding has important implications for media campaigns. It suggests that a spokesperson with whom the audience identifies insures the greatest likelihood of achieving lasting attitude or behavior change.

1. Bhutada NS, Menon AM, Deshpande AD, Perri M. Impact of celebrity pitch in direct-to-consumer advertising of prescription drugs. Health Mark Q. 2012 Jan;29(1):35-48. doi:10.1080/07359683.2012.652576

Online surveys were conducted to determine the impact of endorser credibility, endorser effectiveness, and consumers’ involvement in direct-to-consumer advertising. In a randomized posttest only study, using the elaboration likelihood model, survey participants (U.S. adults) were either exposed to a fictitious prescription drug ad with a celebrity or a noncelebrity endorser. There was no significant difference in credibility and effectiveness between the celebrity and the noncelebrity endorser. High involvement consumers viewed the ad more favorably and exhibited significantly stronger drug inquiry intentions during their next doctor visit. Further, consumers’ involvement did not moderate the effect of celebrity endorser.

1. Brown WJ, Basil MD, Bocarnea MC. The influence of famous athletes on health beliefs and practices: Mark McGwire, child abuse prevention, and androstenedione. J Health Commun. 2003 Feb;8(1):41-57. doi:10.1080/10810730305733.

When Mark McGwire broke Roger Maris's home run record in September of 1998, he was instantly declared an American hero and held up as a positive role model for teenagers and young adults. The extensive media attention focused on McGwire made the general public aware of his use of a muscle-building dietary supplement, Androstenedione. It also increased the public's awareness of McGwire's public service to prevent child abuse. The present research assesses audience involvement with McGwire through parasocial interaction and identification, and the effects of that involvement on audience knowledge of and attitudes toward Androstenedione and child abuse prevention. Results indicate parasocial interaction with an athlete regarded as a public role model likely leads to audience identification with that person, which in turn promotes certain attitudes and beliefs. In this case, parasocial interaction and identification with Mark McGwire was strongly associated with knowledge of Androstenedione, intended use of the supplement, and concern for child abuse. Implications of this research for featuring celebrities in health communication campaigns are discussed.

1. Erdogan BZ, Baker MJ, Tagg S. Selecting celebrity endorsers: the practitioner’s perspective. J Advertising Res. 2001 Jun;41(3):39-48.

Although a number of scholars have investigated effective celebrity endorser characteristics with consumer samples using experimental methods, there is only one study by A. R. Miciak and W. L. Shanklin (1994) that explored the point of view of practitioners who are responsible for the selection of celebrities. This paper investigates British advertising agency managers' consideration of important celebrity characteristics (trustworthiness, expertise, physical attractiveness, familiarity, and likability) when selecting an endorser and these factors' importance according to product types. The research findings validate much of the consumer-based research in that managers consider a range of criteria when choosing celebrity endorsers and indicate that the importance of the criteria depends on the product type.

1. Erdogan BZ, Drollinger T. Endorsement practice: how agencies select spokespeople. J Advertising Res. 2008 Dec;48(4):537-82. doi:10.2501/S0021849908080549.

This research explores how an advertising agency selects a celebrity endorser. The study was conducted in two phases: in-depth interviews that helped the researchers have a greater understanding of the underlying process and a survey mailed to advertising agencies to better examine the steps of the model and test various hypotheses. A normative model of celebrity endorser selection was proposed as a guide for academics and practitioners alike. The model potentially can be useful at each step of the celebrity-endorser selection process.

1. Frizzell C. Public opinion and foreign policy: the effects of celebrity endorsements. Soc Sci J. 2011 Jun;48(2):314-23. doi:10.1016/j.soscij.2010.12.002.

Celebrities often use their popularity to advance international causes, but do they have an effect on public opinion regarding foreign policy? This study uses an experimental method to test whether a statement by Bono of the rock band U2 regarding a fictional foreign policy crisis influences public opinion more than a traditional political elite. Experimental participants were less likely to support the specific position advocated and less likely to be interested in following the crisis in the news when the statement came from Bono, though the results are dependent on party identification and how the scenario was framed.

1. Kim Y, Na J. Effects of celebrity athlete endorsement on attitude towards the product: the role of credibility, attractiveness and the concept of congruence. International Journal of Sports Marketing & Sponsorship. 2007 Jul;8(4):310-20.

This research addresses how the fit between celebrity athlete endorsers and the endorsed products may influence product attitudes. The findings reveal that participants evaluated an endorsed product more favourably when the fit between the celebrity athlete endorser and the endorsed product was congruent (vs incongruent; Experiment 1). Furthermore, participants in the high concept of congruence condition evaluated the endorsed product more favourably than those in the low concept of congruence condition only when the fit between the celebrity athlete endorser and the endorsed product was incongruent (Experiment 2).

1. McCracken, G. Who is the celebrity endorser? Cultural foundations of the endorsement process. J Consum Res. 1989 Dec;16(3):310-21.

Proposes an alternative to explanations for celebrity endorsement based on source credibility (C. I. Hovland and W. Weiss; see record 1953-03515-001) and source attractiveness (W. J. McGuire, 1985) models. The meaning transfer model proposes that celebrities' effectiveness as endorsers stems from cultural meanings with which they are endowed. This model shows how meanings pass from celebrity to product to consumer.

1. Miller FM. Laczniak GR. The ethics of celebrity-athlete endorsement: what happens when a star steps out of bounds? J Advert Res. 2011 Sep;51(3):499-510.

Celebrity athletes are a mainstay of popular culture and an increasingly important part of the marketing ecosystem. As product endorsers, they can influence brand attitudes and sales but also have broader societal implications for the firm. The recent string of bad behavior by celebrity athletes raises important ethical questions about firms that use the famous and infamous to endorse branded products. The conceptual framework presented in the current study provides a theoretical approach—based on virtue ethics—for evaluating the retention of tainted celebrity affiliates. This framework is applied to three well-known situations to examine the ethical implications of what initially were good choices for firms, their brands, and their consumers. The overarching goal of this article is to stimulate managers to think more deeply about the interconnections between their core company values, the athlete endorsers they select, and the ultimate effect of those decisions on their brands in the marketplace if things go wrong.

1. Pornpitakpan C. The effect of celebrity endorsers' perceived credibility on product purchase intention: the case of Singaporeans. J Int Consum Market. 2003;16(2):55-74. doi:10.1300/J046v16n02_04.

This research examines the effect of three celebrity credibility dimensions (attractiveness, trustworthiness, and expertise) on purchase intention with 880 Singaporean undergraduates, using four Asian celebrities as stimuli. In contrast to the results in Ohanian (1991; see also record 1991-26094-001), which indicate that the dimensions attractiveness and trustworthiness do not affect product purchase intention, the results in this study show that all of the three credibility dimensions positively relate to purchase intention. Explanations for the different findings and managerial implications are discussed.

1. Pringle H, Binet L. How marketers can use celebrities to sell more effectively. J Consum Behav. 2005 Mar;4(3):201-14. doi:10.1002/cb.2.

The first part of this paper summarises the key points about the use of celebrities in advertising, sets this particular creative technique in context and demonstrates how significant its return on investment can be. In the second part the paper goes on to report a more detailed analysis of the 'celebrity' case histories among the winners in the IPA Effectiveness Awards, and how practitioners have applied celebrity use to brands to make exceptional impacts on profitability.

1. Reeves RA, Baker GA, Truluck CS. Celebrity worship, materialism, compulsive buying, and the empty self. Psychol Market. 2012 Sep;29(9):674-9. doi:10.1002/mar.20553.

This study tested common predictions from the absorption-addiction model of celebrity worship and the empty self theory. A sample of 171 university students completed a set of scales that included celebrity worship, materialism, and compulsive buying, as well as self-concept clarity, and several other measures of well-being, such as boredom proneness, self-esteem, and life satisfaction. As predicted, materialism and compulsive buying were significantly correlated with celebrity worship, extending research on the empty self theory. Celebrity worship, materialism, and compulsive buying were significantly related to lower self-concept clarity and to lower levels of well-being, supporting both absorption-addiction and empty self theories. The results provide clear evidence for absorption-addiction and empty self theory predictions of a compromised identity. Implications for future research were discussed.

1. Spry A, Pappu R, Cornwell TB. Celebrity endorsement, brand credibility and brand equity. Eur J Marketing. 2011;45(6):882-909. doi:10.1108/03090561111119958.

PURPOSE: This research aims to examine the impact of celebrity credibility on consumer-based equity of the endorsed brand. The mediating role of brand credibility and the moderating role of the type of branding (parent versus sub-brand) employed by the endorsed brand on the endorser credibility-brand equity relationship are also to be examined. The endorser credibility-brand equity relationship was developed using associative learning principles whereas the brand signalling theory is applied to examine the mediating role of brand credibility. DESIGN/METHODOLOGY/APPROACH: The conceptual framework was tested using a field experiment. Data were collected using a mall-intercept approach at a shopping centre from a sample of consumers in a metropolitan Australian city. The data were analysed using structural equation modelling. FINDINGS: Results suggest endorser credibility has an indirect impact on brand equity when this relationship is mediated by brand credibility. This mediating relationship was moderated by type of branding. However, the "endorser credibility-brand credibility" and "endorser credibility-brand equity" relationships did not vary according to the type of branding employed. PRACTICAL IMPLICATIONS: In support of past findings, the present study shows that a celebrity endorser should be one who is perceived as credible based on their attractiveness, expertise and trustworthiness. Moreover, in this research, even a moderately low credibility endorser proved to be able to build the brand. ORIGINALITY/VALUE: The present study is one of the first to empirically examine and confirm the impact of endorser credibility on brand credibility and consumer-based brand equity.

1. Stallen M, Smidts A, Rijpkema M, Smit G, Klucharev V, Fernández G. Celebrities and shoes on the female brain: The neural correlates of product evaluation in the context of fame. J Econ Psychol. 2010 Oct;31(5):802-11. doi:10.1016/j.joep.2010.03.006.

Celebrity endorsement is omnipresent. However, despite its prevalence, it is unclear why celebrities are more persuasive than (equally attractive) non-famous endorsers. The present study investigates which processes underlie the effect of fame on product memory and purchase intention by the use of functional magnetic resonance imaging methods. We find an increase in activity in the medial orbitofrontal cortex (mOFC) underlying the processing of celebrity–product pairings. This finding suggests that the effectiveness of celebrities stems from a transfer of positive affect from celebrity to product. Additional neuroimaging results indicate that this positive affect is elicited by the spontaneous retrieval of explicit memories associated with the celebrity endorser. Also, we demonstrate that neither the activation of implicit memories of earlier exposures nor an increase in attentional processing is essential for a celebrity advertisement to be effective. By explaining the neural mechanism of fame, our results illustrate how neuroscience may contribute to a better understanding of consumer behavior.

1. Till BD, Stanley SM, Priluck R. Classical conditioning and celebrity endorsers: an examination of belongingness and resistance to extinction. Psychol Market. 2008 Feb;25(2):179-96. doi:10.1002/mar.20205.

Three studies attempt to better explain how celebrities are used effectively as conditioned stimuli in the associative learning process. Study 1 establishes that direct affect transfer can occur using celebrities via conditioning. Study 2 suggests that celebrity conditioning will be more effective when there is an appropriate fit (belongingness) between the celebrity and the product endorsed--also known as the match-up hypothesis. Finally, Study 3 examines whether attitudes toward brands paired with celebrities are resistant to efforts to extinguish them using extinction procedures. The findings suggest that conditioning with celebrities yields brand attitudes that are robust and enduring.

1. Veer E, Becirovic I, Martin BAS. If Kate voted conservative, would you? The role of celebrity endorsements in political party advertising. Eur J Marketing. 2010;44(3-4):436-50. doi:10.1108/03090561011020516.

PURPOSE: This research has been conducted with the aim of determining if celebrity endorsers in political party advertising have a significant impact on UK voter intentions. The use of celebrity endorsements is commonplace in the USA, but little is known about its effects in the UK. This research also aims to incorporate the use of celebrity endorsements in political party advertising with the political salience construct. Political salience represents how prominent politics and political issues are in the minds of the eligible voter. DESIGN/METHODOLOGY/APPROACH: A 2 (endorser: celebrity; non-celebrity)×2 (political salience: high; low) between-subjects factorial design experiment was used. The results show that celebrity endorsements do play a significant role in attitudes towards the political advert, attitudes towards the endorser and voter intention. However, this effect is significantly moderated by political salience. FINDINGS: The results show that low political salience respondents were significantly more likely to vote for the political party when a celebrity endorser is used. However, the inverse effect is found for high political salience respondents. PRACTICAL IMPLICATIONS: The results offer significant insights into the effect that celebrity endorsers could have in future elections and the importance that political salience plays in the effectiveness of celebrity endorsement. If political parties are to target those citizens that do not actively engage with politics then the use of celebrity endorsements would make a significant impact, given the results of this research. ORIGINALITY/VALUE: This research would be of particular interest to political party campaigners as well as academics studying the effects of advertising and identity salience.

1. Wei P, Lu H. An examination of the celebrity endorsements and online customer reviews influence female consumers’ shopping behavior. Comput Hum Behav. 2013;29(1):193-201. doi:10.1016/j.chb.2012.08.005.

The goal of this study is to compare the influence of celebrity endorsements to online customer reviews on female shopping behavior. Based on AIDMA and AISAS models, we design an experiment to investigate consumer responses to search good and experience good respectively. The results revealed that search good (shoes) endorsed by a celebrity in an advertisement evoked significantly more attention, desire, and action from the consumer than did an online customer review. We also found that online customer reviews emerged higher than the celebrity endorsement on the scale of participants’ memory, search and share attitudes toward the experience good (toner). Implications for marketers as well as suggestions for future research are discussed.

1. Wheeler RT. Nonprofit advertising: impact of celebrity connection, involvement and gender on source credibility and intention to volunteer time or donate money. Journal of Nonprofit & Public Sector Marketing. 2009 Jan;21(1):80-107. doi:10.1080/10495140802111984.

Two studies examine celebrity endorsers in a nonprofit context. In Study One a framework is developed incorporating connection (congruence), source credibility, involvement, and gender as key elements in understanding the impact of celebrity endorsers on nonprofit advertising effectiveness. Hypotheses are tested in the course of the studies that manipulate celebrity connection and advertising involvement. Results support the primary model viewing a relationship between the celebrity’s connection, source credibility, and intention. Study Two verifies the connection and source credibility findings of Study One, but cannot confirm the impact of the celebrity connection on intention. Study Two includes attractiveness as a source credibility dimension, and increases the number of endorser types. An attractiveness main effect on intention is identified.

1. Zhang H, Liu X. How deeply involved should celebrities be in advertising? Acta Psychol Sin. 2010 May;42(5):587-98.

The advertising literature indicates that celebrity endorsement can facilitate brand awareness and reinforce purchase intention. If the image of a specific celebrity fits well with that brand, the effect of endorsement can be strengthened through meaning transfer or emotional attachment. However, little is known about how deeply the celebrities should be involved in the advertising. While celebrity endorsement has been shown to be able to influence the perception and behavior of customers, to what extent should celebrities be involved in the advertising, and how would this affect the relationship between the brand and customers? Would the celebrity involvement be contingent on product categories and contexts? The authors tried to answer these questions by conducting a 2 (involvement: high and low) x 2 (product category: hedonic and utility) x 2 (need for cognition: high and low) experiment. In the pretest, 57 subjects were recruited from a prestigious university in China. Later on, 140 students from the two universities were divided into four subgroups to participate in the experiment, and 120 responses were used for analysis. The ANOVA results indicated that involvement could significantly affect both customer attitudes toward a brand and their perception of its brand culture. Higher celebrity involvement in the advertising could positively improve customers' attitude and their evaluation of the brand's underlying cultural values, and this effect existed for both hedonic and utility products. In addition, need for cognition played a moderating role in the relation between involvement and customer attitude. This study enriches the literature on celebrity endorsement by introducing the concept "celebrity involvement" in advertising research. The findings show that, for both hedonic and utility products, high levels of celebrity involvement in the advertising lead to positive impacts on customer attitude and perception of brand culture. Therefore, if a firm hopes to make full use of celebrity endorsement, it will be insufficient to use only his/her image or name in the advertising. Instead, the firm should carry out well-organized marketing campaigns to cultivate the deeper meanings and values of this celebrity so as to build up closer relation between customers and the brand.

**Culture Database**

***Humanities Abstracts, 1984-2013 (166 retrieved, 2 relevant and unique)***

1. Alperstein NM. Imaginary social relationships with celebrities appearing in television commercials. J Broadcast Electron. 1991;35(1):43-58. doi:10.1080/08838159109364101.

A variety and range of imaginary social relationships with celebrities appearing in television commercials were explored using ethnographic interviews and self−reflective reports of 60 informants. Interpretation of the descriptions suggests that viewers’ responses to celebrity appearances in television commercials go beyond emulation toward pseudo-social interactions. These interactions can provide great meaning to a viewer involved in an imaginary social relationship with a celebrity appearing in a commercial. This research demonstrates that even when viewers are skeptical of the advertisement, they may reach beyond the intended message where the confluence of information, gossip, and prior exposure to the celebrity converge. This activity becomes part of the individual's social construction of reality.

1. Choi SM, Rifon NJ. Who is the celebrity in advertising? Understanding dimensions of celebrity images. J Pop Cult. 2007 Apr;40(2):304-24. doi:10.1111/j.1540-5931.2007.00380.x.

A study was conducted to identify celebrity image dimensions and develop a scale for measuring them. The development of the scale occurred in three phases: experts generated an extensive list of potential items for the scale, the factor structure of the scale was tested with a group of students from a large Midwestern university, and, finally, the scale was further refined, and its dimensionality validated and finalized. Four distinct dimensions of celebrity image emerged, namely genuineness, competence, excitement, and sociability. Each of these factors or dimension reflected certain traits or characteristics that describe a celebrity's image.

1. Olson IR, McCoy D, Klobusicky E, Ross LA. Social cognition and the anterior temporal lobes: a review and theoretical framework. Soc Cogn Affect Neurosci. 2013 Feb;8(2):123-33. doi:10.1093/scan/nss119.

Memory for people and their relationships, along with memory for social language and social behaviors, constitutes a specific type of semantic memory termed social knowledge. This review focuses on how and where social knowledge is represented in the brain. We propose that portions of the anterior temporal lobe (ATL) play a critical role in representing and retrieving social knowledge. This includes memory about people, their names and biographies and more abstract forms of social memory such as memory for traits and social concepts. This hypothesis is based on the convergence of several lines of research including anatomical findings, lesion evidence from both humans and non-human primates and neuroimaging evidence. Moreover, the ATL is closely interconnected with cortical nuclei of the amygdala and orbitofrontal cortex via the uncinate fasciculus. We propose that this pattern of connectivity underlies the function of the ATL in encoding and storing emotionally tagged knowledge that is used to guide orbitofrontal-based decision processes.

1. Prabhakaran R, Thompson-Schill SL. The price of fame: the impact of stimulus familiarity on proactive interference resolution. J Cogn Neurosci. 2011 Apr;23(4):816-31. doi:10.1162/jocn.2010.21501.

Interference from previously learned information, known as proactive interference (PI), limits our memory retrieval abilities. Previous studies of PI resolution have focused on the role of short-term familiarity, or recency, in causing PI. In the present study, we investigated the impact of long-term stimulus familiarity on PI resolution processes. In two behavioral experiments and one event-related fMRI experiment, long-term familiarity was manipulated through the use of famous and nonfamous stimuli, and short-term familiarity was manipulated through the use of recent and nonrecent probe items in an item recognition task. The right middle frontal gyrus demonstrated greater sensitivity to famous stimuli, suggesting that long-term stimulus familiarity plays a role in influencing PI resolution processes. Further examination of the effect of long-term stimulus familiarity on PI resolution revealed a larger behavioral interference effect for famous stimuli, but only under speeded response conditions. Thus, models of memory retrieval--and of the cognitive control mechanisms that guide retrieval processes--should consider the impact of and interactions among sources of familiarity on multiple time scales.

1. Ross LA, Olson IR. What's unique about unique entities? An fMRI investigation of the semantics of famous faces and landmarks. Cereb Cortex. 2012 Sep;22(9):2005-15. doi:10.1093/cercor/bhr274.

Famous people and artifacts are referred to as "unique entities" (UEs) due to the unique nature of the knowledge we have about them. Past imaging and lesion experiments have indicated that the anterior temporal lobes (ATLs) as having a special role in the processing of UEs. It has remained unclear which attributes of UEs were responsible for the observed effects in imaging experiments. In this study, we investigated what factors of UEs influence brain activity. In a training paradigm, we systematically varied the uniqueness of semantic associations, the presence/absence of a proper name, and the number of semantic associations to determine factors modulating activity in regions subserving the processing of UEs. We found that a conjunction of unique semantic information and proper names modulated activity within a section of the left ATL. Overall, the processing of UEs involved a wider left-hemispheric cortical network. Within these regions, brain activity was significantly affected by the unique semantic attributes especially in the presence of a proper name, but we could not find evidence for an effect of the number of semantic associations. Findings are discussed in regard to current models of ATL function, the neurophysiology of semantics, and social cognitive processing.

**Sociology Database**

***Sociological Abstracts, 1952-2013 (194 identified, 32 relevant and unique)***

1. Ball S, Eckel CC. The economic value of status. J Soc Econ. 1998;27(4):495-514.

Academic works on status from a variety of fields are reviewed, & a theory of status in economics -- ie, status-seeking occurs because increased status leads to economic benefits -- is presented. The influence of status on human behavior is examined in a "box market" experiment using 69 undergraduates, some of whom were given "star" status, supposedly based on quiz results, but really by chance; star status was revealed to all subjects (Ss). An additional bargaining ultimatum game experiment, in which proposer Ss were given a choice between dividing $10 in either a high-pay or low-pay fashion, while respondent Ss could accept or reject the proposal, is detailed. It was found that star respondents were treated more generously in the proposals than nonstar respondents. A series of market experiments is also presented with star status assigned randomly & with all Ss being aware of this randomness. Despite the apparent meaninglessness of star status, results were similar to those in the first experiment. It is concluded that status changes expectations about what outcomes are reasonable in economic games.

1. Barrow AE 3rd. The effect of celebrity athlete models in food advertising on the perceived healthiness of food products [master’s thesis]. [Norman (OK)]: University of Oklahoma; 2003. 96 p.

Recognizing the seriousness of the obesity problem, the government, consumer groups and parents have put pressure on the food industry to adjust their advertising practices (Seiders & Petty, 2007). The use of certain celebrity athletes in advertisements is particularly concerning as they may be seen as "healthy" role models. Therefore, empirical research is important to understand the potential impact that the presence of the celebrity athlete model poses to food choices. The purpose of this study is to determine how the use of a celebrity athlete model in food advertising affects the perception of healthiness of the advertised food product among college students. The methodology for this study is a simple experimental design using an experimental (treatment) ad and a control ad. Participants were randomly assigned one of three different print ads for a breakfast cereal. One treatment features celebrity endorser Michael Phelps, another treatment features celebrity endorser Eli Manning, and the final treatment features a non-celebrity male model. Findings and conclusions. There were at total of 106 students sampled from Oklahoma State University journalism classes for the questionnaire. The results of the present study indicate that college students in the research sample were not influenced by the presence of a celebrity athlete in food advertising. However, the respondents did rate the ad containing Eli Manning significantly more appealing than the ad containing the control ad and Michael Phelps. This partially confirms what is largely accepted in advertising practices, in that celebrity endorsers do create a more favorable impression of products and brands when they are used in the ads. The fact that Phelps was not rated higher than the control ad in the Attitude toward the Ad scale could largely be due to his recent negative media attention following his drug use scandal.

1. Barry E. Celebrity, cultural production and public life. Int J Cult Stud. 2008 Sep;11(3):251-58. doi:10.1177/1356766908092583.

Despite a continuing increase in the dollar value of athlete endorsement contracts and the prominence of athlete endorsements as a marketing tool, the value of endorsement contracts has gone largely unexamined. Employing event study analysis, this paper assesses the effects of endorsement contract announcements on changes in the share price of firms. In contrast to previous studies which focus on a single megastar athlete or sporting event and find significant positive returns to the firm, this study evaluates 148 endorsement announcements for conventional athletic stars in various sports and finds that the average endorsement contract has an insignificant impact on the market value of the firm. Also, there is no support of the product-endorser match-up hypotheses but endorsements by golfers do exhibit significant abnormal returns.

1. Basil MD. Identification as a mediator of celebrity effects. J Broadcast Electron. 1996 Oct;40(4):478-95.

An analysis of the role of consumer identification on the effectiveness of celebrity endorsers. Replicating a study conducted by M. D. Brown & M. D. Basil (1995 [see abstract 9604409]) directly after Ervin "Magic" Johnson's announcement that he had tested positive for human immunodeficiency virus, questionnaire data from 147 undergraduates at the U of Hawaii, Manoa, were used to measure the effectiveness of the announcement on acquired immune deficiency syndrome-related attitudes, beliefs, behaviors, & identification one year after the announcement. Results, similar to the previous study, showed that gender & TV reliance were significant predictors, & personal concern & intention to change high-risk behavior were significant identification effects. Path analysis demonstrated that identification with Johnson mediated effects on perception & behavior. Larger issues surrounding the effectiveness of media campaigns on health behavior are discussed, along with policy implications.

1. Bezbaruah N. Impact of using celebrity spokes-characters to market fruits and vegetables to parents and children [dissertation]. [Fargo (ND)]: North Dakota State University of Agriculture and Applied Sciences; 2010.

The prevalence of overweight and obesity especially among children has become a major public health issue. Overweight children and adolescents are at greater risk for health problems during their youth and as adults. Diets with low-energy density and high nutrient content have been associated with less weight gain. A strategy to prevent weight gain in children and adolescents is to increase consumption of fruits and vegetables since most fruits and vegetables are foods that have high nutrient density. Advertising and marketing play a crucial role in the preference and consumption of food and beverages, especially for children. Therefore, celebrity spokes-characters have been used to market these products to children. There had been some allegations that celebrity spokes-characters had been used to market unhealthy food products to children. Following such allegations, Nickelodeon network has been restricting the use of their licensed characters on packages of non-nutrient dense foods. As part of the new policy celebrity spokes-characters, including Dora the Explorer and Sponge Bob Square Pants, are seen on packages of fruits and vegetables. The move behind this initiative is to encourage children to consume more fruits and vegetables. The main purpose of the present study is to examine the impact of using celebrity spokes-characters to market fruits and vegetables to parents and children. The study included 233 parents and 218 third and fourth grade students from four schools. Survey and experimental study methods were utilized to examine the effect of celebrity spokes-characters on fruit and vegetable intake among children. Family likes and dislikes was the leading factor that determined purchasing decisions of parents/guardians in regard to fruits and vegetables. In an experimental study the presence of a celebrity spokes-character significantly increased intake of green beans among children. Children ranked taste as the leading factor that was considered in choosing whether to consume fruits and vegetables or not.

1. Biccum A. Marketing development: celebrity politics and the “new” development advocacy. Third World Q. 2011 Aug;32(7):1331-46. doi:10.1080/01436597.2011.600107.

Politics and culture, once considered separate, are now fusing in new and interesting ways. Political activism is becoming popular, particularly through the expansion of a new kind of development advocacy made highly visible through celebrity involvement. Theorists of globalisation celebrate the democratisation of civil society made possible by new information and communications technology; critical theorists will note the various ways in which it ambivalently makes the contradictions in global capitalism more obvious and has become the means by which globalisation is contested. Some metropolitan governments have sought to capitalise on this new knowledge economy by making knowledge for development part of their strategies to produce "global citizens" necessary for the global economy. This paper examines the linkages between celebrity and government-funded development advocacy in Australia, which comprise the introduction of free market principles to form a marketing campaign for neoliberal globalisation.

1. Brown WJ, Basil MD. Media celebrities and public health: responses to “Magic” Johnson’s HIV disclosure and its impact on AIDS risk and high-risk behaviors. Health Commun. 1995;7(4):345-70.

The public announcement of his human immunodeficiency virus (HIV) status by Earvin "Magic" Johnson in 1991 was intended to promote prevention, particularly among adolescents & young adults, the largest population of his fans. Examined here are public responses to this announcement as they relate to perceptions of HIV awareness & risk. Questionnaire data were gathered from 391 students at a US university, 10 days after Johnson went public, to assess personal involvement with the star prior to the announcement, the role of media exposure, & changes in attitude about risky behaviors. Results indicate that those with an emotional involvement with Johnson were more likely to show an increased awareness of HIV & an intention to increase safe-sex practices, though simply knowing of the star had no measurable impact on any of these factors. Suggestions for the use of celebrities in similar promotional campaigns are provided.

1. Ding H, Molchanov AE, Stork PA. The value of celebrity endorsements: a stock market perspective. Market Lett. 2011 Jun;22(2):147-63. doi:10.1007/s11002-010-9117-y.

Are celebrity endorsements worthwhile investments in advertising? To answer this question, we analyze a unique sample of 101 announcements made between 1996 and 2008 by firms listed in the USA. Internet is the main medium of communication for these announcements. We employ event study methodology and document statistically insignificant abnormal returns around the announcement dates. This finding is consistent with the notion that the incremental benefits from celebrity endorsements closely match the incremental costs due to such contracts. Further, we investigate if the announcement date return depends on a number of characteristics that are often used in the endorsement literature. As a result, we find that endorsements of technology industry products coincide with significant positive abnormal returns around the announcement dates. Finally, we find weak support for the match-up hypothesis between celebrities and endorsed products.

1. Dix S, Phau I, Pougnet S. ‘Bend it like Beckham’: the influence of sports celebrities on young adult consumers. Young Consum. 2010;11(1):36-46. doi:10.1108/17473611011025993.

Purpose: The purpose of this paper is to investigate how sports celebrities can be perceived as role models and influence young adult consumers' purchase and behavioural intentions. Further, it also seeks to examine whether this influence differs between males and females. Design-methodology-approach: A self-administered questionnaire was designed using established scales. A convenience sample was drawn from students in a large university in Western Australia. Findings: Athlete role model endorsers have a positive influence on young adults' product switching behaviour, complaint behaviour, positive word-of-mouth behaviour and brand loyalty. This confirms the assumption that sports celebrities are important socialisation agents and can have significant impact on purchase intentions and behaviours. Practical implications: This research provides useful insight into the influence of athlete endorsers on young adults and suggests athletes have a positive influence on young adults' behavioural intentions in switching products, generating word-of-mouth and establishing brand loyalty. More importantly, this study is a significant step towards providing useful information about how young consumers respond to the use of sports celebrities in advertising. Originality-value: Previous studies indicate that this potential influence and impact of sports star endorsers would be at its peak amongst the youth market. This paper extends previous studies by focusing on one specific market - young adults in Australia.

1. Farrell N. Celebrity politics: Bono, Product (RED) and the legitimising of philanthrocapitalism. Br J Polit Int Relat. 2012 Aug;14(3):392-406. doi:10.1111/j.1467-856X.2011.00499.x.

This article reviews a framework developed by John Street which positions aesthetics, style and performance, and celebrity politics as legitimate features within representative democracy. It applies this framework to the example of (RED), a political consumerism campaign fronted by U2 singer Bono, which raises funds for African AIDS victims. It accounts for the use of style by Bono as a celebrity politician to represent himself as an authoritative figure and (RED) as a legitimate response to the epidemic, and relates this representation to the organisational arrangements underpinning the campaign. Further, it examines the relationship between the interests of these organisations and the manner in which (RED) represents AIDS. The article argues for a further integration of textual readings of celebrity politicians based on their aesthetic qualities, and an appreciation of the organisations that contribute to the production of their campaigns.

1. Ferris KO. The sociology of celebrity. Sociol Compass. 2007 Sep;1(1):371-84.

The sociology of celebrity (and its cousin, fame) is a relatively young field, despite having identifiable classical roots. While the topic was ignored by sociologists for many years, it has recently been taken up by both theorists and empirical researchers in sociology and a variety of related fields. In this article, I evaluate the current state of the field, and identify two major themes - celebrity as pathology and celebrity as commodity - that currently dominate the literature. In addition, I suggest additional research directions that I believe will help the field develop and mature; in particular, empirically grounded and meaning-oriented research that reflects the lived experiences of those who swim in the sea of celebrity culture everyday. What does celebrity mean to the people who produce it, consume it, engage with it and live it? To the extent that researchers take up these questions, the sociology of celebrity will continue to be a vibrant and vigorous area of study.

1. Fizel J, McNeil CR, Smaby T. Athlete endorsement contracts: the impact of conventional stars. Int Adv Econ Res. 2008 May;14(2):247-56. doi:10.1007/s11294-008-9144-0.

Despite a continuing increase in the dollar value of athlete endorsement contracts and the prominence of athlete endorsements as a marketing tool, the value of endorsement contracts has gone largely unexamined. Employing event study analysis, this paper assesses the effects of endorsement contract announcements on changes in the share price of firms. In contrast to previous studies which focus on a single megastar athlete or sporting event and find significant positive returns to the firm, this study evaluates 148 endorsement announcements for conventional athletic stars in various sports and finds that the average endorsement contract has an insignificant impact on the market value of the firm. Also, there is no support of the product-endorser match-up hypotheses but endorsements by golfers do exhibit significant abnormal returns.

1. Frizzell C. Public opinion and foreign policy: the effects of celebrity endorsements. Soc Sci J. 2011 Jun;48(2):314-23. doi:10.1016/j.soscij.2010.12.002.

Celebrities often use their popularity to advance international causes, but do they have an effect on public opinion regarding foreign policy? This study uses an experimental method to test whether a statement by Bono of the rock band U2 regarding a fictional foreign policy crisis influences public opinion more than a traditional political elite. Experimental participants were less likely to support the specific position advocated and less likely to be interested in following the crisis in the news when the statement came from Bono, though the results are dependent on party identification and how the scenario was framed.

1. Hambrick ME, Mahoney TQ. ‘It’s incredible - trust me’: exploring the role of celebrity athletes as marketers in online social networks. Int J Sport Manag Market. 2012;10(3-4):161-79. doi:10.1504/IJSMM.2011.044794.

Sport marketers have acknowledged the popularity of online social networks, but have struggled with transforming them into viable revenue generators. Using celebrity athletes to promote products via online social networks such as Twitter may represent one potential revenue opportunity. This study examined how Lance Armstrong and Serena Williams used Twitter for promotional purposes and utilised content analysis to analyse their combined 7,202 Twitter messages. The celebrity athletes wrote promotional messages 12% of the time, primarily to promote their corporate sponsors and products, charitable organisations and personal activities. Twitter represents a unique marketing resource and communication channel for celebrity athlete product endorsements.

1. Hewer P, Brownlie D. Spaces of hope, enlivenment and entanglement: explorations in the spatial logic of celebrity culinary brands. J Consum Cult. 2013 Mar;13(1):46-63. doi:10.1177/1469540512474531.

This article explores the production of the 'Nigella' celebrity brand through forms of gendered talk performed by means of online community forums. The complexity and appeal of celebrity culinary brands forces us to turn to particular contexts to explore the passions, concerns and enthusiasms that they elicit and excite. As a context for the exploration of such hyper-mediated brands it is useful to explore the social interactions and associations harboured and sheltered within the collective canopy of the forum, in our case the Food Forum of Nigella.com. The emotional fabric of celebrity culinary brands has much to do with the fact that they are created and sustained through a range of multimedia platforms. One such critical stage is that of the online forum, which we explore as a site wherein feminine identities are performed and reimagined; where notions of 'doing gender' within culinary landscapes are worked and reworked through networks of affiliation and shared sentiment.

1. Hewer P, Hamilton K. Exhibitions and the role of fashion in the sustenance of the Kylie Brand mythology: unpacking the spatial logic of celebrity culture. Market Theory. 2012 Dec;12(4):411-25. doi:10.1177/1470593112457737.

Central to the logic of the aesthetic economy (Entwistle, 2002) is celebrity culture, the two go hand in hand and the boundaries between them are increasingly blurred in the spirit and name of commerce, capitalism and marketing. Celebrity demands a stage, or better, an exhibition space, a frame for further performance and sustainment of appeal, a space to induce inspiration among followers and fans alike. Celebrity by this reckoning is less about advertising and the transfer of meaning through such overly narrow and confined media processes (McCracken, 1989); rather celebrity affect, as we seek to demonstrate in this paper, works through specific stagings, one such being the celebrity fashion exhibition, where the appeal of celebrity is broadened and staged anew to cultivate new forms of attraction, intimacy and public participation.

1. Hunter D. Celebrity entrepreneurship: communication effectiveness through perceived involvement. Int J Enterpren Small Bus. 2007 Jul 4;4(5):505-27. doi:10.1504/IJESB.2007.014387.

Increasingly, celebrities appear not only as endorsers for products but are apparently involved in entrepreneurial roles in the ventures that market the products they promote. We call this phenomenon Celebrity Entrepreneurship. We hypothesise that celebrity entrepreneurs are more effective communicators than typical celebrity endorsers. Further, we hypothesize that this is because celebrity entrepreneurship leads to higher perceptions of Involvement an endorser quality hitherto neglected in the marketing communication literature which in turn affects traditional outcome variables such as Aad and Abr. Two experiments confirm that a) involvement can successfully be operationalised as distinct from variables previously shown to influence communicator effectiveness, b) involvement has a positive effect on Aad and Abr over and above the traditional predictors, and c) celebrity entrepreneurship in experimental manipulations leads to increased perceived involvement.

1. Hyman MR, Sierra JJ. Idolizing sport celebrities: a gateway to psychopathology? Young Consum. 2010;11(3):226-38. doi:10.1108/17473611011074296.

Purpose: Sport celebrities often endorse their team, their sport, and non-sports-related products. Increased idolizing of sport celebrities by adolescents is one artifact of this promotional practice. Although seemingly innocuous, adolescents who idolize sport celebrities may, as adults, come to worship such celebrities; this unhealthy obsession may afflict 10 percent or more of adults. If adolescent hero worship of sport celebrities is a gateway to this adult psychopathology, then alerting parents, as well as encouraging social responsibility among advertisers and sport teams/leagues, is critical. This paper aims to address the issues. Design/methodology/approach: After a brief review of the literature on adolescent hero worship, the literature on the determinants and effects of celebrity worship are explored. Findings: Once parents, advertisers, sport team/leagues are sensitized to the problem, adolescent hero worship of sport celebrities can be mitigated as a likely gateway to many adults' unhealthy obsession with celebrities. Research limitations/implications: Directions for future sport celebrity worship research are suggested. Practical implications: The incidence of a potentially psychologically damaging affliction can be reduced without harm to advertisers, sport teams/leagues, and athletes. Social implications: Ways to reduce promotion-induced sport celebrity worship - without eliminating sport promotion per se - are suggested. Recommendations are targeted for sport-related and non-sport-related products as well as teams and leagues/conferences. Originality/value: This paper is the first to suggest a link between adolescent hero worship of sport celebrities and psychologically dangerous celebrity worship by adults.

1. Jackson DJ, Darrow TIA. The influence of celebrity endorsements on young adults’ political opinions. Harv Int J Press-Pol. 2005 Jul;10(3):80-98. doi:10.1177/1081180X05279278.

This research contributes to our understanding of the increasing mixture of entertainment and politics by examining the impact of the political statements made by celebrities on the opinions of Anglophone Canadian youth. A recent survey of young Canadians enrolled in first-year university political science courses indicates young people's level of agreement with certain political statements is increased by the endorsement of these positions by Canadian celebrities from the realms of popular music and sports. These results suggest that celebrity endorsements make unpopular statements more palatable, while increasing the level of agreement with already popular opinions.

1. Jain V, Roy S, Daswani A, Sudha M. What really works for teenagers: human or fictional celebrity? Young Consum. 2011;12(2):171-83. doi:10.1108/17473611111141623.

Purpose: This study aims to explore the relative effectiveness of a human celebrity endorser vis-a-vis a fictional celebrity or character endorser on teenage consumers' attitudes. Further, the study also seeks to assess whether the effectiveness varies depending on the nature of the product being endorsed. Design/methodology/approach: Given the purpose of the study, experimental design was used as the research methodology. In an experimental set-up three product categories (low-involvement food/low-involvement non-food/high-involvement) and two endorsers (human celebrity/fictional celebrity) and a control group were deployed in a 33 full factorial design on 378 teenagers. Fictitious advertisements were used as stimuli. Findings: The study suggests that, for food and non-food low-involvement product categories, the impact of a human celebrity is more than that of a fictional celebrity. Regarding the purchase intentions of teenagers, it was found that a human celebrity is more effective than a fictional celebrity in food and non-food low-involvement products. In the case of the high-involvement product, the human celebrity was not found to create favorable consumer attitudes. Research limitations/implications: The study results suggest that celebrity endorsements are useful, but the nature of the product also has an influence on success. One limitation of the study was the restriction to print advertisements. Practical implications: A major implication from the findings for the managers is that a human celebrity may not always be the right choice for any product promotion for teenagers. More specifically, for high-involvement products, celebrity endorsement needs to be handled with caution since it may not prove to be successful. Originality/value: The contribution of the study is in addressing an area that has not been very well researched as yet, and in addressing a research question that has not been investigated properly.

1. Limbu YB, Huhmann BA, Peterson RT. An examination of humor and endorser effects on consumers' responses to direct-to-consumer advertising. Int J Pharmaceut Healthc Market. 2012;6(1):23-38. doi:10.1108/17506121211216888.

Purpose: This study aims to examine how product involvement moderates the effects of emotional appeals namely humor and endorsers on consumers' responses to direct-to-consumer advertising (DTCA). Design/methodology/approach: This study employed a 2 (Humor: humor vs. non-humor) 2 (Endorser: celebrity vs expert) 2 (Involvement: high vs low) factorial experimental design. Subjects were 420 allergy/asthma sufferers or non-sufferers attending a large Southwestern US university as undergraduate and graduate students. Findings: Results confirm that low involvement consumers demonstrate more positive responses than high involvement consumers toward prescription drug ads with emotional appeals. Humor or a celebrity endorser enhances ad and brand attitudes, brand recall, and copy point recall of consumers without medical conditions. However, an expert endorser is found to be more effective in improving ad credibility. A three-way interaction between humor, endorser, and involvement was evident indicating that the celebrity endorser and humor jointly generated more positive responses than other combinations of treatment group when product involvement was low. These findings clearly suggest that use of emotional appeals in DTCA does not influence attitudes and memory of target audience who are suffering from a condition. Originality/value: This is the first empirical study that examines the effects of emotional appeals namely humor and endorsers on consumers' responses to DTCA.

1. Lindenberg S, Joly JF, Stapel DA. The norm-activating power of celebrity: the dynamics of success and influence. Soc Psychol Quart. 2011 Mar;74(1):98-120. doi:10.1177/0190272511398208.

On the basis of previous evidence, we reasoned that even if people do not identify with celebrities, these celebrities can influence their behavior by activating bundles of social norms. Activating a norm means making both content and "oughtness" of the norm more directly relevant for behavior. We further reasoned that in order to have this norm-activating effect, celebrities have to have prestige. The question is whether they need to be seen as successful in order to have this effect. In four experimental studies, we examined the effects of a normative message presented by a celebrity on the activation of a target norm and of related and unrelated norms. As predicted, the normative message activated both target and related norms and did not activate unrelated norms. Also as expected, this ability to activate norms vanished entirely when the celebrities were tarnished by waning success. This result also shows that "success" and "lack of success" of a celebrity can be the result of relatively minor differences in media reporting. As expected, the norm-activating effect of celebrities was not mediated by self-reported measures of seeing celebrities as role models or of identifying with them. Implications for the impact of someone's environment on norm conformity beyond positive and negative sanctions are discussed.

1. Makgosa R. The influence of vicarious role models on purchase intentions of Botswana teenagers. Young Consum. 2010;11(4):307-19. doi:10.1108/17473611011093934.

Purpose: The purpose of this paper is to investigate how vicarious role models such as television celebrities and entertainers influence purchase intentions of teenagers in Botswana. Design/methodology/approach: A survey research design was used. Data were collected from a convenience sample of 200 senior secondary school students using a structured questionnaire. The measures used in the questionnaire were adapted from previous scales. Findings: The results reflected that there is a relatively high level of vicarious role model influence of television celebrities and entertainers among teenagers in Botswana. Similarly, the results of regression analysis demonstrated that television celebrities and entertainers as vicarious role models positively influence teenagers' purchase intentions, especially their switching behavior and response to the problem. Research limitations/implications: By examining how vicarious role model influence of television celebrities and entertainers affects the purchase intentions of Botswana teenagers, this study has not only studied a cultural context that has not been investigated before but also enriches the existing body of research on how young consumers acquire purchase-related behavior. Practical implications: Based on the findings of the current study, marketers could safely use vicarious role models such as celebrities and entertainers when designing television advertisements aimed at the teenagers segment. Originality/value: It is widely held that teenagers learn a significant proportion of their purchase behavior through direct and indirect contact. Hence, various socialization agents that influence teenagers' purchase behavior have been studied using western samples. The paper is one of the few that have contributed knowledge about how the purchase behavior of teenagers in an African context is influenced by television celebrities and entertainers as socialization agents.

1. McDermott ST, Hocking JE, Johnson L, Atkin CK. Adolescents’ responses to sports figure product endorsements. Southern Commun J. 1989 Jul;54(4):350-63.

A school-administered questionnaire survey of 135 adolescents in grades 6, 8, & 10 in rural Ga was conducted in 1988 to assess the relationship between exposure to TV ads containing celebrity endorsements of smokeless tobacco products with attitudes, beliefs, & perceptions about the use of smokeless tobacco. The results suggest that a simple exposure model is not sufficient for explaining the effects of celebrity endorsements. However, use of smokeless tobacco, perceptions of acceptability of use by friends, & positive attitudes toward users were all related to Ss' particular viewing experiences. 2 Tables, 12 References. Adapted from the source document.

1. Moeran B. Celebrities and the name economy. Res Econ Anthropol. 2003;22:299-321.

Drawing on research in the worlds of advertising, magazines, & fashion, this paper discusses how celebrities mediate between different fields of cultural production. By focusing on celebrity endorsements in advertising, it also outlines how film actors & actresses, athletes, models, pop singers, & sportsmen & women mediate between producers & consumers via the products & services that they endorse. As economic mediators, celebrities' actions have important strategic & financial implications for the corporations whose products they endorse. As cultural mediators, they give commodities personalities & perform across different media, linking different cultural fields into an integrated name economy.

1. Muruganantham G, Kaliyamoorthy S. Celebrity endorsement - a competitive tool for brand positioning. Int J Value Chain Manag. 2010;3(4):386-400. doi:10.1504/IJVCM.2009.031768.

One of the biggest challenges in marketing communications is how to break through ever increasing media clutter. With media becoming fragmented, engaging the customer has become difficult. Celebrity endorsements can help to meet this challenge due to the characteristics they offer. India is becoming a celebrity-obsessed society due to increase in celebrity advertisements in various media. The purpose of this paper is to study the positioning strategies of two leading fairness cream brands and to assess the role played by celebrities to position the brand in the minds of the target segment. The results show that users and non-users of the study brands perceive the brand positioning meaning differently. Conclusions and recommendations are presented for those involved in personal care brand development and marketing research.

1. O’Reilly NJ, Braedley LA. Celebrity athletes and athletic clothing design: branding female tennis players. Int J Sport Manag Market. 2008;3(1-2):119-39.

Interest in celebrities and their lifestyles makes the clothing they wear an important part of their image. Due to their influence on markets, celebrity athletes are no longer simply product endorsers; many are now also involved in product design. The present study adopts both expert interviews and observation methodologies to explore the celebrity athlete-clothing relationship in high-profile female tennis players. A content analysis of the 2005 Wimbledon Championships and interviews with experts in both the sport marketing and fashion industries provided for the development of a model that articulates the interactions between the celebrity athlete, the label, the market, and the clothing the athlete wears during competition. Results outline the important connection between celebrity athletes, their clothing and the tennis audience. Applicability to practice and impetus for future research are provided.

1. Quadagno D, Eberstein IW, Foster K, Sittig JE, Sly DF, Kistner JA. Magic Johnson and children's conceptions of AIDS. AIDS Educ Prev. 1997 Aug;9(4):359-72.

Longitudinal data for a heterogeneous sample of 609 elementary school children are used to assess the long-term effects of Magic Johnson's announcement on children's HIV and AIDS conceptions. Four hypotheses are tested concerning these relationships, and background variables measured prior to Johnson's announcement are controlled. Findings suggest that Johnson's announcement increased children's HIV and AIDS knowledge and reduced their prejudice toward a hypothetical child with AIDS. No relationship is evident between the announcement and perceived vulnerability to HIV and AIDS. Males are more likely to be aware of Johnson's announcement, but its effects are more pronounced among blacks. Findings from the present research affirm the potential for celebrities like Johnson in HIV and AIDS education campaigns directed toward children.

1. Rojek C. Sports celebrity and the civilizing process. Cult Sport Soc. 2006 Oct;9(4):674-90. doi:10.1080/17430430600769106.

One of the most important concepts in the figurational/process sociological approach is also one of the most under-examined: functional democratization. This refers to the tendency of functional specialization & the division of labour to diminish imbalances in the power balance between groups. The essay discusses the concept &, drawing on Emile Durkheim's concept of 'egoism', argues that the rise of celebrity culture provides an important challenge for the concept. The Sports Star is now the object of intense emotional attachment from fans. This exceeds an attachment based upon appreciation of sporting prowess. The leading Sports Stars, in common with the leading celebrities from celebrity culture, are adopted as role models by fans & their lives are followed as parables of normative behaviour. A new term, invasive egoism, is introduced to describe the development of fantasy relationships between the fan & the sports star. Invasive egoism can produce over-close identification between fans & their idols, so that the media representations of the star's life become the fulcrum for measuring changes & moments of significance in the life of the fan. The rise of the Sports Star is related to the onslaught of the commodification of sports culture. References. Adapted from the source document.

1. Tyler I, Bennett B. 'Celebrity chav': fame, femininity and social class. Eur J Cult Stud. 2010 Aug;13(3):375-93. doi:10.1177/1367549410363203.

This article argues that celebrity is an increasingly significant means by which reactionary class attitudes, allegiances and judgements are communicated. In contradistinction to claims that the concept of social class has lost its analytic value in the context of contemporary consumer society and the growing ideological purchase of meritocracy and choice, the article contends that class remains central to the constitution and meaning of celebrity. A central premise of this article is that celebrity culture is not only thoroughly embedded in everyday social practices, but is more radically constitutive of contemporary social life. This claim is examined through a consideration of the ways in which celebrity produces and sustains class relations. The article argues that a new category of notoriety or public visibility has emerged and is embodied in the figure of the working-class female celebrity within celebrity culture and wider social life.

1. Wheeler M. The democratic worth of celebrity politics in an era of late modernity. Br J Polit Int Relat. 2012 Aug;14(3):407-422. doi:10.1111/j.1467-856X.2011.00487.x.

In a seminal article published in 2004, John Street argued that celebrity politics has provided a greater expression for the enhancement of democratic behaviour. Consequently, this analysis builds on Street's thesis to consider the worth of celebrity politics in an era of late modernity. To this end, it employs Henrik Bang's and John Keane's constructs of Everyday Makers and Monitory Democracy, which have emphasised the importance of 'involvement', 'voice' and 'output' in terms of representation, to provide an ideological framework to capture the value of celebrity politics. Subsequently, it may be argued that Barack Obama utilised a form of 'liquid' celebrity in his 2008 US presidential campaign to reconnect with a disenfranchised electorate. However, this article will critically assess these types of celebrity politics to contend that aggregated forms of 'input' drawn from celebrity activism may more truly affect political outcomes.

1. Zauner A, Fink M, Maresch D, Aschauer E. Community marketing in social media - can marketers leverage Facebook groups of celebrities? Int J Enterpren Small Bus. 2012;16(4):406-21. doi:10.1504/IJESB.2012.047609.

Social media networks (e.g., Facebook) have become a major factor influencing various aspects of consumer behaviour and have thus also become the targets of marketers. Social networks that gather special interest groups, such as celebrity fan groups, provide a particularly attractive point of leverage for community marketing. However, there remain open questions regarding the success of community marketing activities in social media. The present study attempts to close this gap by investigating whether: 1) the image of the brand and 2) the celebrity endorser credibility of a top sports team influence the perceived customer value of the sponsoring firm. Based on a survey of members of a Facebook fan group of an Austrian celebrity sports team (n = 322), we identify a direct positive impact of celebrity-centred community marketing in social media on customer value. Our findings emphasise the relevance of coordinated marketing activities that use social media to create customer value.
